# Photosensitizers-Loaded Nanocarriers for Enhancement of Photodynamic Therapy in Melanoma Treatment

**DOI:** 10.3390/pharmaceutics15082124

**Published:** 2023-08-11

**Authors:** Ana Maria Udrea, Adriana Smarandache, Andra Dinache, Catalina Mares, Simona Nistorescu, Speranta Avram, Angela Staicu

**Affiliations:** 1 Lasers Department, National Institute for Lasers, Plasma and Radiation Physics, 409 Atomistilor Str., 077125 Magurele, Romania; ana.udrea@inflpr.ro (A.M.U.); andra.dinache@inflpr.ro (A.D.); simona.stroescu@inflpr.ro (S.N.); 2 Department of Anatomy, Animal Physiology and Biophysics, Faculty of Biology, University of Bucharest, 91-95 Splaiul Independentei, 050095 Bucharest, Romania; catalina.sogor@gmail.com (C.M.); speranta.avram@gmail.com (S.A.)

**Keywords:** melanoma, photodynamic therapy, photosensitizer, porphyrins, non-porphyrin photosensitizers, nanocarriers

## Abstract

Malignant melanoma poses a significant global health burden. It is the most aggressive and lethal form of skin cancer, attributed to various risk factors such as UV radiation exposure, genetic modifications, chemical carcinogens, immunosuppression, and fair complexion. Photodynamic therapy is a promising minimally invasive treatment that uses light to activate a photosensitizer, resulting in the formation of reactive oxygen species, which ultimately promote cell death. When selecting photosensitizers for melanoma photodynamic therapy, the presence of melanin should be considered. Melanin absorbs visible radiation similar to most photosensitizers and has antioxidant properties, which undermines the reactive species generated in photodynamic therapy processes. These characteristics have led to further research for new photosensitizing platforms to ensure better treatment results. The development of photosensitizers has advanced with the use of nanotechnology, which plays a crucial role in enhancing solubility, optical absorption, and tumour targeting. This paper reviews the current approaches (that use the synergistic effect of different photosensitizers, nanocarriers, chemotherapeutic agents) in the photodynamic therapy of melanoma.

## 1. Introduction

With more than 1.5 million new cases diagnosed and over 120,000 skin-cancer-associated deaths reported in 2020, skin tumours are the most commonly diagnosed group of cancers worldwide [1]. Out of these, the most frequent cases belong to the epidermal (non-melanoma) skin cancer [2]. These are the least aggressive and rarely lethal skin tumours that predominantly occur in the region subjected to extreme sun exposure. Although significantly less common (5% of cutaneous malignancies) than non-melanoma skin cancers, malignant melanoma is nonetheless the most aggressive one, being responsible for about 80% of overall skin cancer deaths due to poor therapeutic prognosis [2,3]. Melanoma neoplasm develops from melanocytes in the skin, mucosa, or uvea [4]. Exposure to UV radiation that can induce DNA damage [5,6], chemical carcinogens [7], genetic modification [4], fair skin with blue eyes and red hair [8], and immunosuppression [9,10] are some of the recognized risk factors for melanoma.

Current therapeutic strategies for melanoma include surgical treatments, systemic therapy (chemotherapy, including new targeted compounds and immunotherapeutic drugs), and adjuvant therapy (radiotherapy, photodynamic therapy (PDT), immune therapy, immune checkpoint inhibitors, molecular-targeted therapy, chemotherapy), depending on the clinical stage and the risk grade of patients [11,12,13,14,15,16,17]. However, the heterogeneity and the significant predisposition of these tumours for metastatic spreading are responsible for their poor response to conventional therapies. Therefore, the development of new therapeutic agents or alternative or combined strategies that can overcome this resistance and lack of response is needed.

Photodynamic therapy (PDT), a clinically accepted treatment procedure, involves the use of a photosensitizer (PS) and a specific wavelength of light to selectively destroy abnormal or cancerous cells in the body. This is a promising therapy option for melanomas mainly due to its minimally invasive nature, low risk of side effects and systemic toxicity, lack of resistance to repeated treatments, as well as good aesthetic outcome [17,18,19,20]. Moreover, PDT can be used alone or in combination with other therapeutic modalities, including radiotherapy, chemotherapy, surgery, gene therapy, and immunotherapy [21]. It has been the subject of in-depth research and became recognized as a disease-site-specific therapeutic method since its conception at the end of 19th century when Niels Finsen was awarded the Nobel Prize in Physiology or Medicine (in 1903) for his contribution to the field of phototherapy. In the same period, Von Tappeiner and A. Jesionek described the treatment of skin tumours using a combination of light and the organic dye eosin. The process, which Von Tappeiner called ‘photodynamic action’, led to modern PDT [22]. A significant breakthrough occurred in 1978, when Thomas Dougherty and co-workers reported that the administration of hematoporphyrin derivative (HpD) followed by local exposure to red light resulted in complete or partial response in 111 of 113 cutaneous or malignant lesions, malignant melanoma included [23]. Since then, the advancement of PDT has had numerous milestones, including the approval of several PDT medications for various tumours, and clinical trials for new PDT strategies are ongoing [24,25].

Over the last years, nanotechnology-based PDT has emerged as a promising approach for treating cancer. This procedure involves either directly modifying PSs using nanotechnology or delivering PSs through nanocarriers, which enhances their ability to target specific tumour sites and improves the effectiveness of PDT for cancer treatment, including various types of skin cancer, melanoma, squamous cell carcinoma, basal cell carcinoma, and actinic keratosis [26,27].

This study provides an extensive and detailed examination of the current advancements in using nanocarriers based on porphyrin and non-porphyrin photosensitizers for the photodynamic therapy of malignant melanoma. By systematically reviewing the state-of-the-art research, this study offers valuable insights into the potential benefits and challenges associated with this innovative approach. Through a meticulous review of different nanocarrier formulations, we highlight their versatility and effectiveness in delivering therapeutic agents to melanoma tumours. This targeted approach is important in achieving better treatment outcomes and reducing potential side effects, making it a promising avenue for future melanoma therapies. 

## 2. The Main Elements of PDT and Melanoma

### 2.1. The Principles of Photodynamic Therapy

**Figure 1:**
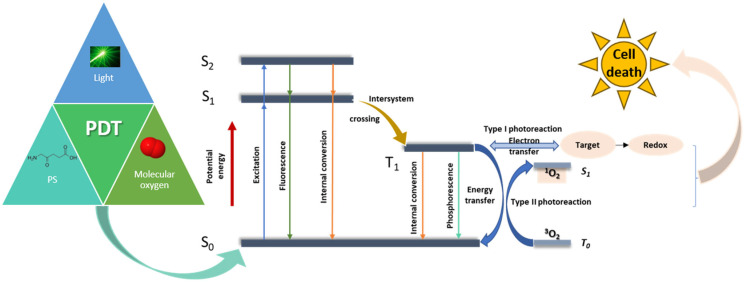
The Jablonski diagram illustrating PDT mechanism of action with the physical processes leading to Type I and Type II reactions, which may eventually result in oxidative cell damage. S_0_ is the ground state of the photosensitizer (PS); S_1_ and S_2_ are the first and, respectively, the second excited singlet states of PS; T_1_ is the first excited triplet state of PS; *T*_0_ is the ground state of triplet oxygen; ^3^O_2_, triplet oxygen; *S*_1_ is the excited state of singlet oxygen; ^1^O_2_, singlet oxygen.

PDT involves three main elements: light, a PS, and molecular oxygen (Figure 1). The PSs are non-toxic in the absence of light, but when exposed, they can trigger a chain reaction of photo-chemical processes that produce cytotoxic reactive oxygen species (ROS), which promote cell death [28]. Therefore, when activated by light at a proper wavelength, PS molecules undergo a transition to an excited energetic state S_1_. In this state, the PS has a very short lifetime (in the order of ns), and it may return to the ground state by emitting fluorescence or via a non-radiative decay. As an alternative, through intersystem crossing, the PS can have a transition from a singlet to a triplet state T_1_, which has a considerably longer lifetime. A PS in its triplet state can likewise return to the ground state in a similar manner by emitting phosphorescence or releasing heat energy. Furthermore, a PS in a triplet excited state can directly interact with cellular substrates through electron transfer, which results in the generation of free radicals. Thus, PSs react with oxygen and yield ROS such as superoxide anion radicals, O_2_^٠−^, hydroxyl radicals, ˙OH, and hydrogen peroxides, H_2_O_2_ (Type I reaction). PSs can also transfer energy to triplet oxygen in the ground state (^3^O_2_) through a Type II reaction to generate highly reactive singlet oxygen, ^1^O_2_. In the end, the reactive species produced during the photodynamic process destroy cancer cells directly through apoptosis, necrosis, or autophagy, as well as indirectly by injuring the tumour vascularity, which causes tumour ischemia [29]. It is important to note that both Type I and Type II reactions can occur at the same time. The ratio between these processes is influenced by the nature of the PS, as well as the concentrations of O_2_ molecules. However, the experimental studies show that photoactivated ^1^O_2_ generation, specifically via the Type II reaction, dominates in PDT [28].

The skin’s optical properties are important issues that should be taken into account when choosing the appropriate PSs for the PDT of melanoma. Melanocytes, which are present in the basal layer of the epidermis, are responsible for skin pigmentation and melanin formation, which protects against harmful UV radiation. Melanin can significantly affect treatment efficacy due to its light-absorbing properties and antioxidant capabilities. The quantity and distribution of this chromophore determine the light transport through the skin. The absorption coefficient of melanin is $\mu_{a}^{melanin}=\left(5\times 10^{9}\right)\lambda^{3.33}$ cm^−1^, where λ refers to the wavelength expressed in nm [30]. The origin of the optical properties of natural melanin is still a difficult problem to solve and continues to be a significant barrier to understanding the complex photoprotective role played by this natural pigment. For the diagnosis of skin conditions, the dosimetry of laser radiation used in phototherapy, and the synthesis of photoactive compounds, knowledge of the optical properties of melanin is required [31,32,33]. The UV-Vis absorption spectrum of in situ melanin (in human skin) highlights the gradual rise in absorbance values from 750 to 600 nm, followed by a moderate rise from 600 to 450 nm and, finally, a sharply rise from 450 nm to a broad peak at around 335 nm, below which it gradually decreases to much lower values [34]. Thus, melanin competes with PS absorption in the same spectral range and decreases the efficacy of PDT. PSs that absorb in the near-infrared (NIR) are consequently better for the PDT of melanoma because of the optical window in this range in biological tissue, and consequently, a deeper penetration of light is attained [35]. Researchers also found different strategies to overcome this drawback [36], and such an approach was reported by Freitas and his colleagues, where gold NPs irradiated with NIR radiation, tuneable optics and photothermal properties can exert synergistic effects with PSs in PDT [37].

Melanin is also considered an intracellular antioxidant; thus, it neutralizes PDT-induced ROS and decreases treatment success [38]. Melanin-related DNA damage can be lessened by PDT’s singlet oxygen, which can also reduce the natural oxidation of melanin [39]. The ability of melanin to scavenge ROS, including singlet oxygen, hydroxyl radicals, and superoxide anions, has been reported. These studies suggest that melanin protects pigmented cells from oxidative stress, alters cell metabolism, triggers immunological suppression, and causes mutagenesis of the tumour microenvironment, protecting malignant melanocytes from different therapeutic approaches [40]. Depigmentation has received extensive attention as a technique to overcome PDT resistance in melanoma. [35]. In order to reduce the pigment level of melanotic melanoma, several strategies were proposed by research teams in the field. Gomaa et al. use phenylthiourea (PTU) as a melanin synthesis inhibitor. PDT via exposure to 56.2 J/cm^2^ of monochromatic red laser emitted at 652 nm has been applied on depigmented melanoma cells using liposomes-encapsulated sodium ferrous chlorophyllin (Fe-CHL), resulting in LC_50_ values of 18.20 and 1.77 μM after 24 and 48 h incubation. The mechanism of cell death of Fe-CHL-mediated PDT was found to be a combination of both apoptosis and necrosis [41].

### 2.2. Photosensitizers

**Figure 2:**
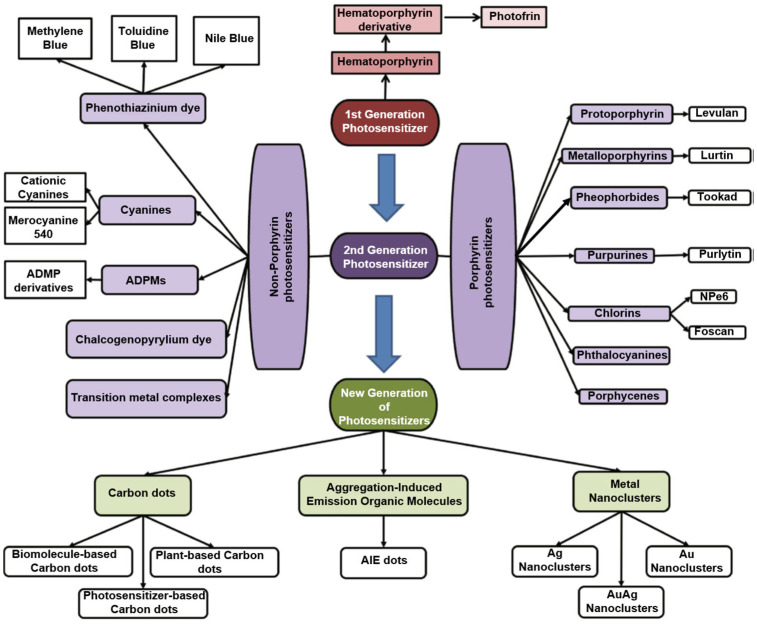
A schematic evolution of PSs reproduced from ref. [43], where ADPM represents Azadipyrromethene, NPe6 is mono-L-aspartyl chlorin e6, and AIE denotes Aggregation-Induced Emission.

There are several types of photosensitizers, including (i) porphyrin-type PSs such as porphyrins, chlorins, bacteriochlorins, and phthalocyanines; (ii) non-porphyrin PS dyes, such as phenothiazinium salts, Rose Bengal, squaraines, boron–dipyrromethene (BODIPY) dyes, phenalenones, and transition metal-complex dyes; and (iii) naturally occurring compounds such as perylenequinones, flavins, and curcuminoids [42]. Figure 2 shows the evolution of different generations of PSs, as reported by Tavakkoli Yaraki et al. [43].

A first generation of porphyrins were introduced as PSs in treatments for the first time in the 1970s by Dr. T. Dougherty and his collaborators [23,44]. They were tested a water-soluble porphyrin, HpD, which was synthesised via the purification and chemical modification of hematoporphyrin (Hp). HpD possesses better tissue selectivity for tumours and is less aggressive with the skin when compared to Hp. Subsequently, a mixture of porphyrin dimers and oligomers isolated from HpD was available under the trade name Photofrin^®^, which is currently the most commonly used PS [42,45]. However, first-generation PS applications in PDT have some limitations as a consequence of their low chemical purity or poor tissue penetration due to maximum light absorption in the visible spectral range. In addition, some side effects can occur after PDT, such as skin hypersensitivity to light for several weeks because of the long half-life of the PS and its high accumulation in the tissues. Other disadvantages include photobleaching and low absorption at wavelengths higher than 600 nm. 

These drawbacks of the first-generation PSs led to research on new compounds and initiated the development of the second generation of PSs with the possibility of NIR activation and high production of ^1^O_2_ beginning in 1980 [45,46]. NIR radiation has minimal interaction with surrounding biological components, hence affording increased tissue penetration depth and high biomedical imaging resolution [47]. 

Hundreds of substances with potential photosensitizing properties have been proposed as second-generation PSs, but not as many reached clinical trials. The number of compounds officially approved for clinical use in anticancer PDT is even more limited [48]. Typically, these are macrocyclic complexes derived from substitutions of the porphyrin moieties or direct modifications of the porphyrin core, or some new non-porphyrin PS molecules [46,49], including metalloporphyrins (Lutrin^®^ and Lutex^®^, Pharmacyclics, Sunnyvale, CA, USA), porphycenes, pheophorbides (Tookad^®^, ImPact Biotech, Ness Ziona, Israel)**,** purpurins (Purlytin^®^, Miravant Medical Technologies, Gaviota, CA, USA), phthalocyanines, chlorins (Foscan^®^, Biolitec Pharma, Jena, Germany), protoporphyrin IX precursors (Hexvix^®^, Photocure Oslo, Norvegia, Metvix^®^, Galderma Laboratories, Fort Worth, TX, USA, and Levulan^®^, Sun Pharma, Mumbai, India), phenothiazines (methylene blue, and toluidine blue), cyanines, dipyrromethenes, hypericin, and xanthene dyes (Rose Bengal) [28,50]. 

A synthesis of the main classes of first- and second-generation PSs, their activation wavelengths, and their quantum yields of singlet oxygen generation, which are PSs’ main characteristics, is given in Table 1. Compared to the first generation, second-generation photosensitizers have better photostability and light absorption at longer wavelength and, in this way, a higher tissue penetration, higher quantum yield in ROS generation, and greater tumour selectivity [46,51,52].

In PDT applications, chlorins surpass porphyrins, as they exhibit strong absorption at longer wavelengths (650–690 nm), a higher molar absorption coefficient, deeper tumour tissue penetration, low toxicity, and good photostability [53]. Also, second-generation PS, phthalocyanines have multiple benefits, including light absorption at longer wavelengths, homogeneity, a high extinction coefficient, and high quantum yield in ROS generation. The main drawbacks of these PSs are the tendency of their aromatic ring to aggregate and their hydrophobic nature. Derivatives generated by macrocycle modification and/or conjugation with peptides, liposomes, polyethylene glycol, and NPs improve efficiency by increasing biodisponibility, while the NPs disrupt their tendency to aggregate [54,55]. Some of the second-generation porphyrin-based PSs exhibit dark toxicity, and overall, their synthesis and chemical adjustments are challenging. This last disadvantage made researchers show interest in exploring non-porphyrin photosensitizers, which can be produced with greater ease [45].

**Table 1:** Activation wavelengths and quantum yields of PSs.

	PDT Agents	Activation Wavelength(nm)	Quantum Yield/ Solvent	Reference
Porphyrins	Hematoporphyrins	350–420, 620–650	0.63–0.69/methanol 0.5–0.85/ethanol	[56,57]
	Protoporphyrins	405, 500–505, 630–635	0.58–0.92/methanol 0.56–0.67/ethanol	[57,58,59]
	Metalloporphyrins	400–435, 500–520,550–560, 590–630	0.46–0.59/water 0.36–0.48/chloroform	[60,61]
	Pheophorbides	750–790	~1/acetone ~0.5/ micelle solution	[62]
	Purpurins	350–440, 500–550, 600–700	0.4–0.82/methanol or MeOD	[63]
	Benzoporphyrins	360–500, 550–600, 670–700	0.85/DMF 0.53/DMSO	[64,65]
	Chlorins	380–420, 480–550, 590–660	0.43–0.74/pyridine	[66,67]
	Phthalocyanines	350–400, 600–700	0.13–0.67/DMSO 0.12–0.62/ water/methanol	[66,68]
	Porphycenes	350–400, 550–600, 630–730	0.28–0.36/toluene 0.21–0.28/ D2O/pluronic	[69,70]
Non-porphyrins	Squarines	550–600, 650–800	0.005–0.021/toluene	[71,72]
	Cyanines	750–900	0.007–0.169/DCM	[73,74]
	Xanthenes	500–570	0.75/water	[75]
	Phenothiazines	620–700	0.22/water 0.52/DMF	[76]
	Curcuminoids	420–580	0.11/toluene/acetonitrile	[77,78]
	Boron–dipyrromethene (BODIPY)	500–580	~0.2/toluene	[79,80]

Non-porphyrin PSs have shown promise in melanoma treatment, offering unique advantages and facing specific challenges. Among the cyanine class, Indocyanine Green (ICG) exhibits good solubility in water and a strong absorption peak at 800 nm, allowing for deeper tissue penetration and enhanced tumour targeting. However, ICG’s short circulation half-life and rapid excretion are significant drawbacks. Also, it has a relatively low quantum yield in oxygen singlet generation [81]. Rose Bengal (RB), a xanthene dye, is a hydrophilic anionic sensitizer with better solubility in aqueous media, potentially accumulating in melanoma cells. It efficiently generates ROS upon light activation, leading to apoptosis in melanoma cells. Still, it has some disadvantages like limited tumour accumulation and poor selectivity [82,83,84]. Curcumin, a natural compound with low toxicity and various beneficial effects, exhibits poor bioavailability and reduced skin photosensitivity, limiting its potential as a PS [84]. Hypericin (Hyp) efficiently generates ROS upon light activation and may possess tumour selectivity due to its hydrophobic characteristics, allowing it to diffuse through plasma membranes. However, its limited tissue penetration, bioavailability, and hydrophobicity leading to self-aggregation are significant drawbacks [85]. Overall, non-porphyrin PSs have advantages in melanoma treatment, but careful consideration of their limitations is essential for their successful implementation in PDT. 

However, the fundamental flaw of the second-generation PSs is their non-specific localization to targeted cells/tissues and their poor solubility in water, which is a significant limiting factor in their intravenous administration and requires looking for novel drug delivery techniques [48]. Therefore, the attention was turned to designing the targeted third generation of PS. The synthesis of compounds with a higher affinity for tumour tissue, which reduces impact on surrounding healthy tissues, constitutes the basis for this third generation of PSs. The third-generation PSs are characterized by the conjugation of second-generation PSs with targeting entities or moieties or by encapsulation into carriers to improve the accumulation of PS at the targeted tumour sites [86].

Nanotechnology can solve difficulties such as limited solubility, optical absorption, and tumour-targeting capabilities [21,87,88]. Drug delivery nanosystems seem to be a promising treatment in cancer due to the high surface-area-to-volume ratio of the nanostructures which allow drugs to be encapsulated or bound to nanoparticles (NPs) [89]. NP applications within cancer PDT systems are quickly becoming effective due to the NPs’ simplicity to synthesize and simple surface chemistry with high capacity for functionalization. Also, NPs’ small sizes enable their cell internalization, reducing lymphatic filtration and increasing drug uptake, particularly in tumour cells, because of the enhanced permeability and retention effect (EPR) [90]. 

There were over 350 clinical trials registered with the National Cancer Institute (NCI) in 2022 that focused on treating advanced and/or metastatic melanoma using innovative approaches, including immune cell therapy, cancer vaccines, and targeting new therapeutic avenues. Moreover, researchers have developed novel delivery systems utilizing biomaterials in conjunction with approved drugs. As a result, these advancements have the potential to reduce treatment toxicity and boost treatment effectiveness. Among the emerging advanced delivery systems, nanoparticles and liposomes have gained attention for their ability to improve drug stability and prolong systemic circulation time, thus holding promise as effective tools in melanoma treatment [91].

The enclosure of PSs into a nanocarrier system has been most frequently proposed to solve the problems of stability and biocompatibility. As a drug-delivery system, NPs may represent a better delivery method that reduces side effects, has better tumour targeting, and a lower treatment resistance [92]. To improve PDT efficacy, NP platforms are used to passive or active PS delivery in tumour cells.

Passive PS absorption and accumulation in cancer cells is a result of NP composition and size, their overall uptake is only impacted by the tumour environment (such as hypoxia or low pH) and also by the EPR effect [93]. NPs can be categorized into organic, inorganic and hybrids, according to the material type [92]. Micelles and liposomes, polymeric and lipidic nanocarriers, nanoemulsions, dendrimers, metal oxides, ceramics, silica, metal–organic framework (MOF) nanoparticles, and carbon nanotubes are a few examples of nanoplatforms that passively improve PS accumulation in PDT [94,95,96]. 

In active absorption, through a molecular recognition process, the PS is delivered to a particular tumour target. In order to improve the uptake of the PS, the NPs are functionalized with specific ligands that attach to receptors overexpressed by cancer cells. These targeting vehicles include monoclonal antibodies; antibody fragments; peptides; proteins such as transferrin, epidermal growth factor, and insulin; LDL; various carbohydrates; somatostatin; folic acid; and many others [96].

**Figure 3:**
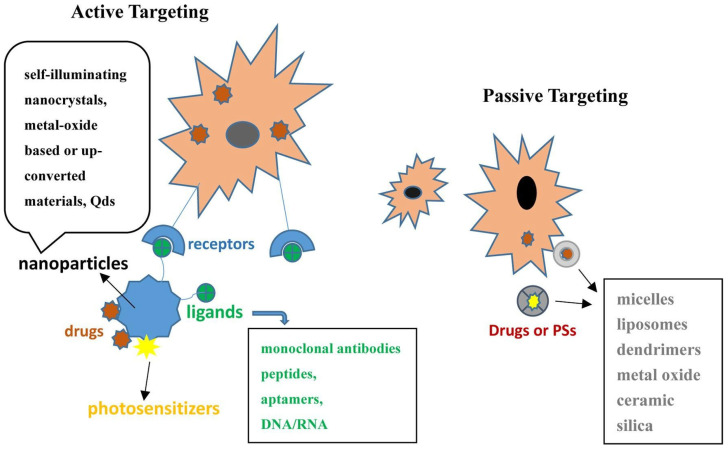
Active and passive forms of PSs or chemotherapeutics in combination with nanocarriers in melanoma PDT reproduced from ref. [36].

Figure 3 synthesizes the means through which NP platforms can be functionalized to improve PS delivery according to Zhang et al.’s review paper [36].

Inorganic nanomaterials like quantum dots, self-illuminating nanocrystals, metals, or metal-oxide NPs are used for the development of platforms that actively increase targeted PS delivery in PDT [97]. 

Silver nanoparticles are promising candidates for melanoma treatment due to their attractive properties, including their antioxidant, antiproliferative, anti-inflammatory, antibacterial, antifungal, and antitumoral capabilities. Their use in combination with PDT, photothermal therapy (PTT), or chemotherapy are new strategies for melanoma treatment [98]. Combining antibody technology and silver nanoparticles could enhance the selectivity and delivery of therapeutic agents for melanoma treatment [99]. Furthermore, metallic NPs can serve as potential tools for cancer detection, via magnetic resonance imaging, and as colloidal intermediaries for magnetic hyperthermia in cancer treatment [100].

**Figure 4:**
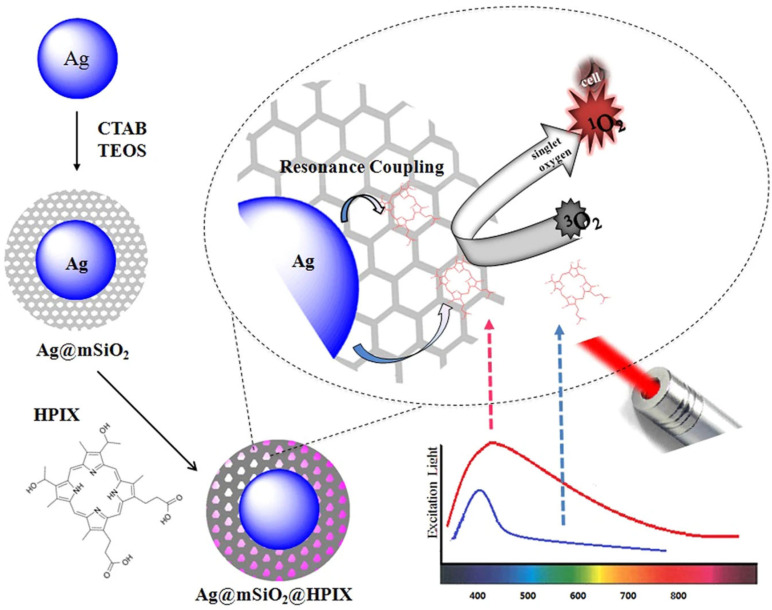
Schematic of the synthesis of Ag@mSiO_2_@HPIX hybrid and its PDT antitumoral action [101].

Plasmonic nanostructures, such as gold or silver, can also be synthesized to have their plasmon absorption in the NIR window, which makes them suitable for local effects even after they are injected into the body [101]. Several morphologies, such as gold nanocage, nanorod-in-shell, and nanoparticle-in-shell, showed strong NIR absorption up to 1100 nm and facilitated the production of ^1^O_2_ to mediate dual-mode PTT and PDT in B16-F10 melanoma tumours [102,103]. A type of hybrid photosensitizer consisting of plasmonic silver nanoparticles coated with mesoporous silica (mSiO_2_) and hematoporphyrin IX (HPIX) was also reported (Figure 4, reproduced from ref. [101]), where strong resonance coupling between the two led to exceptionally high singlet oxygen production under broad spectral excitations. At the dosage level where these hybrid photosensitizers display little cytotoxicity without light illumination, they can effectively inhibit tumoral cells under both visible and red/NIR irradiation. The hybrids showed an increase in singlet oxygen enhancement factor to 4.2, while there was almost no singlet oxygen produced by the free HPIX under 850 nm excitation. 

Presently, most research on PDT advancements relies on two-dimensional (2D) monocultures, which lack the ability to fully replicate the complexity of tissues [104]. Therefore, three-dimensional (3D) cell cultures serve as more suitable models, resembling tumour tissues in terms of their architecture and functionality. The referenced review explores different PS drugs and both passive and active targeted PS nanoparticle-based platforms for PDT treatment of melanoma in both 2D and 3D models. The overall conclusion highlights that very few studies have explored PDT within 3D models using active PS nanoparticle-based platforms, emphasizing the need for further investigation in this area [104].

Synergistic approaches are undertaken, and several studies are reported concerning the use of PDT in combination with other therapies like PTT [105]. Nanosystems act as drug carriers and light absorbents, potentially improving photothermal and photodynamic therapies’ outcomes [25].

Unfortunately, PDT still faces several limitations, such as dealing with metastatic tumours at unknown locations, challenges in the effective delivery of light, and lack of sufficient oxygen. The emergence of new nanomaterials offers a promising approach for delivering multiple therapeutic drugs simultaneously, presenting a potential method for cancer treatment. Utilizing multifunctional nanocarriers to co-deliver two or more drugs can improve their physical and chemical properties, promote tumour site accumulation, and synergistically enhance the antitumor effect [106]. 

Despite the positive outcomes observed with the combination of PDT and chemotherapy, gene therapy, immunotherapy, photothermal therapy, hyperthermia, radiotherapy, sonodynamic therapy, and even multidrug therapy, each approach has its own limitations. PDT has been linked to sustained systemic immunosuppression, yet the underlying mechanism remains unclear. This immunosuppression not only affects the subsequent efficacy of PDT but also impacts the combination of PDT with immunotherapy. Moreover, combining PDT with PTT relies on laser irradiation, which poses challenges for deep-seated tumours. Additionally, PDT’s effectiveness when combined with radiotherapy is influenced by its strong dependence on oxygen. The co-delivery of multiple drugs faces constraints related to delivery vector development, drug loading, and release. These challenges contribute to the limited clinical application of codelivery systems based on nanocarriers. Other factors include the incomplete safety assessment of the preparation protocol and difficulties in large-scale clinical implementation of the approach [106]. Despite several promising in vivo and in vitro studies demonstrating the potential advantages of photodynamic therapy as an adjuvant treatment for melanoma, its clinical application remains restricted due to its relative inefficiency [35]. 

While only a few therapies utilizing nanoparticulate systems have progressed to clinical trials, there is an expectation that a considerable number of these treatments will be adopted for clinical use in the near future. Due to its high sensitivity, specificity, and ability to perform multiplexed measurements, this technology presents significant opportunities for enhancing melanoma treatment. Ultimately, these advancements are expected to lead to improved patient survival rates [25]. 

Finding better PSs that can overcome melanoma resistance is the most significant problem that needs to be solved in order to make PDT a truly effective anti-melanoma treatment. There have been numerous attempts in recent years to address this issue, and the research continues by respecting the following guidelines: (a) the use of PSs that are triggered by light at a higher wavelength, particularly NIR, to prevent melanin absorption and enable deeper tumour penetration, and (b) enhanced tumour targeting and accumulation of the PSs [107].

## 3. Porphyrins

Porphyrins are tetrapyrrole chemical compounds. Their chemical structure includes four pyrrole rings (five-atom rings composed of four carbon atoms and one nitrogen atom) connected by (=(CH)- or -CH_2_- units. Worldwide, porphyrins are used in PDT applications due to their very high stability, however, they exhibit photosensitivity and relatively low tissue penetration [28]. 

Porphyrins are divided into the first and second generations of PSs. Photofrin II is a hematoporphyrin PS and represents the first-generation PSs. This compound has been utilized in PDT to treat different malignancies, such as fibrosarcoma, breast, ovarian, oral, or colorectal cancer. Low light absorption, however, was one of the factors that led to the development of the second generation of PS [51]. The efficacy of Photofrin II in melanoma treatment was investigated and compared with a second-generation PS verteporfin. The study results suggest that the photodynamic effect of verteporfin is 10 times higher than that of Photofrin II [108].

By utilizing cutting-edge techniques and nanotechnology, the third-generation PSs have led to notable improvements in stability, tumour targeting, biodistribution, and activation. Conjugation with specific entities or moieties (antibodies, carbohydrates, amino acids, sugars, folic acid, hormones, peptides) are employed to improve PDT’s tumour targeting and efficacy [86]. The solubility, stability, and pharmacokinetic characteristics of PSs were enhanced via incorporation into nanoscale delivery systems, including NP, micelles, liposomes, or conjugates. Furthermore, these nanoscale platforms provide options for surface functionalization, enabling the attachment of additional therapeutic agents such as doxorubicin cytostatic drug [109].

### 3.1. Porphyrin Derivatives

**Figure 5:**
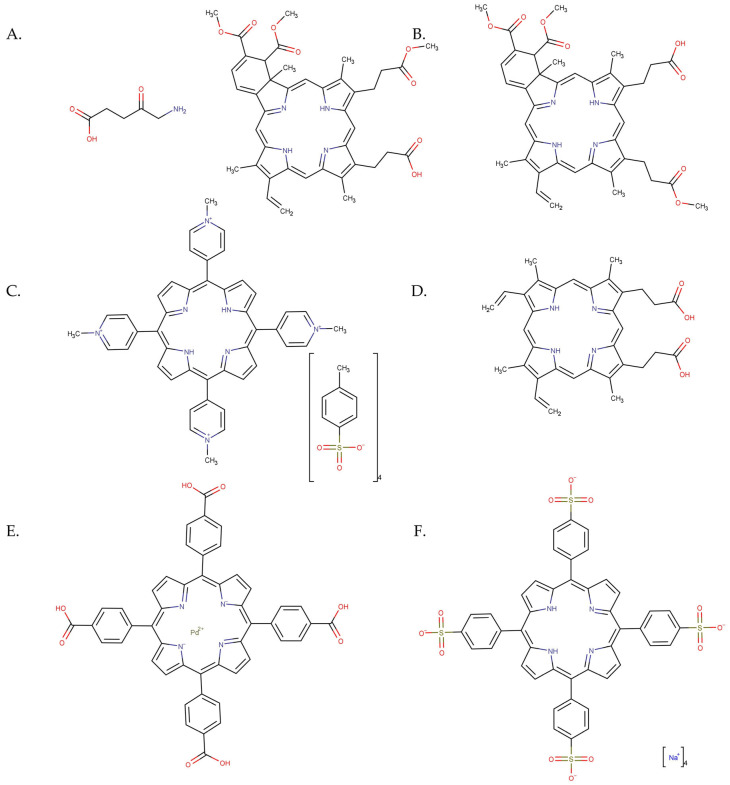
Two-dimensional structures of (**A**) 5-ALA; (**B**) verteporfin; (**C**) 5,10,15,20-(Tetra-N-methyl-4-pyridyl)porphyrin tetratosylate; (**D**) PpIX; (**E**) palladium-meso-tetra (4-carboxyphenyl) porphyrin; (**F**) (5,10,15,20-(Tetra-4-sulfonate phenyl) porphyrin tetraammonium.

The porphyrins synthesis pathway, which produces HEME in humans and chlorophyll in plants, begins with 5-Aminolevulinic acid (5-ALA) (Figure 5), a non-proteinogenic amino acid that exogenously administrated in cells is converted to PpIX [110,111]. The conversion of 5-ALA in PpIX involves eight enzyme-catalysed steps, of which four occur in the mitochondria and four in the cytosol. Fe^2+^ and the rate-limiting enzyme ferrochelatase are required for the future conversion of PpIX to HEME [112].

In physiologic pH, 5-ALA has low lipid solubility and low passage to the cellular membrane. To increase the selectivity and absorption of 5-ALA, several derivatives, various carrier compositions, and skin permeability enhancers have all been examined as ways to accomplish this goal [113]. Cancerous cell membranes tend to be more negatively charged than normal cells, making it challenging for 5-ALA (which also is negatively charged) to efficiently penetrate the cell membranes. However, this challenge can be addressed using specific strategies [113]. For instance, the positive charge of a nanocarrier (for example, gold nanoparticles or multifunctional hollow mesoporous silica nanoparticles) can readily interact with the negative cell membrane, facilitating more effective absorption of the 5-ALA-conjugate [113,114]. Small molecule additives such as two zinc-bis(dipicolylamine) may also enhance the cellular uptake of 5-ALA. This compound exhibits weak interaction with 5-ALA; instead, it acts as a membrane-active additive, temporarily disrupting the cell membrane, thereby facilitating the permeation of 5-ALA [115]. 

Gold NPs improved the photosensitivity of 5-ALA. The potential of the complex as a reliable PS delivery strategy for PDT was evaluated on the Mel-Rm cell line using the MTT assay. Five treatment groups were made to evaluate the efficacy of the conjugate: the control group, the no-drug group (in which the effect of the laser was evaluated without any treatment), the 5-ALA group, the gold NP group, and the complex group. To test the photosensitivity of the conjugate, a He-Ne laser was employed as a light source at varied fluences (20, 40, 60, and 80 J/cm^2^). The results indicate that the best outcomes were attained when the complex was irradiated at a fluence of 60 J/cm^2^ (Table 2). 

The higher inhibitory activity of the complex compared with the 5-ALA without the NP indicate that the gold NPs represent an effective transport agent. Furthermore, conjugation with the NP promotes the entry of 5-ALA into the cells, which may contribute to increased PpIX accumulation. The toxicity of the gold NP is the study’s disadvantage. The NP displayed 20% toxicity and inhibited the growth of the Mel-Rm cell line at a concentration of 0.2 mM [113]. Another difficulty with using 5-ALA in PDT is the quick transformation of converted PpIX into HEME (which has no photosensitive activity) caused by the high level of intracellular iron. Moreover, the inhibition of the DNA repair enzyme is also associated with decreased intracellular iron levels. To overcome those issues, Li et al. used deferoxamine to regulate the intracellular iron ions in cancer cells [116]. Deferoxamine is a medication used as an iron chelator, thus it binds to and removes iron that is not bound to proteins (free iron) and iron that is in the process of being transported between transferrin and ferritin, as well as iron stored in hemosiderin and ferritin [117].

To overcome its limited solubility and permeability, 5-ALA was encapsulated in membrane fusion liposomes (MFLs). MFLs act as a nanocarrier for 5-ALA, and deferoxamine could be internalized into tumour cells through membrane fusion, and drugs encapsulated in liposomes can enter the cytoplasm directly. For PDT, a 532 nm laser with 300 mW/cm^2^ irradiance for 5 min was used as a light source. The results show that MFLs improved pharmacological behaviour and increased the 5-ALA and deferoxamine uptake rate and delivery to cancer cells. The drug-loaded MFLs interact with the cancer cell membrane, releasing 5-ALA and deferoxamine straight into the cytoplasm. The liposomal nanomedicines have intracellular iron control and enhanced pharmaceutical behaviour, resulting in an increased 5-ALA photodynamic activity. The in vitro results show that at a 5-ALA concentration of 2 mM/L, the complex reduced the B16-F10 cells’ viability to 20% compared with 49% in the case of 5-ALA alone and 30% in the case of 5-ALA and deferoxamine. In vivo results are correlated with the in vitro ones. The administration of MFLs-5-ALA-deferoxamine in female C57 mice showed the highest tumour mass decrease compared with 5-ALA, deferoxamine, 5-ALA-deferoxamine, and MFLs 5-ALA. The complex combines the inhibition of 5-ALA biotransformation with the reversal of damaged DNA repair via iron ion control, significantly increasing 5-ALA PDT efficiency (Table 2) [116].

Different studies have chosen to use the B16-F10 melanoma cell line to test the efficacy of porphyrin–nanoparticle complexes in PDT. This cell line is a stable malignant melanoma tumour cell line that can be studied in vivo, the tumour cells being injected into organism, thus developing melanoma in a animal model [118]. Thus, Da Silva et al. showed in [119] that PpIX (Table 2) encapsulation in poly (D, L lactic-co-glycolic acid) (PLGA) NPs improved the PDT effect on the melanoma B16-F10 cells at a light dose (1500 mJ/cm^2^ at 630 nm) with only 21.7% viable cells after exposure. Very good phototoxicity values were observed compared with PpIX, but no cytotoxicity was observed in the dark. Regarding the quantum yield of singlet oxygen generation, this was not influenced by the PpIX encapsulation in the PLGA NPs. Overall, the study showed that the nanocarrier platform is a potential delivery system for melanoma skin cancer, since it maintained the photophysical properties of PpIX and has significant in vitro phototoxicity effect against melanoma cells, reducing cell viability ∼80% (7.91 μg/mL PpIX in Nps), and affords safe PDT [119].

On the other hand, the PpIX complex with polysilsesquioxane (PpIX-PSilQ) NPs applied on A375 cells showed the ability to be internalized via endocytosis [120]. An irradiation dose of 24.5 mW/cm^2^ for 20 min, at 630 nm, proved a decrease of about nine times in the IC50 for PpIX (9.4 µM) vs. PpIX-PSilQ NPs (81.2 µM).

Rizzi et al. studied the mesoporous silica nanoparticles (SNPs) conjugated with verteporfin (Ver), and also obtain a useful way to promote drug selectivity and a good phototoxicity of the nanocomplexes [121]. The cytotoxicity was reduced to half for the Ver-SNPs compared with free Ver, which inhibits the cell proliferation to only 30%. The studies involving Ver-MSN were performed comparatively on normal HaCat keratinocyte cell lines, as well as on A375P (a low metastatic melanoma) and SK-MEL-28 (a high metastatic melanoma). NPs absorption was achieved through endocytosis, forming endosomes. Irradiation with red laser light did not affect the proliferation of normal cells treated with Ver-SNPs, but had an inhibitory effect on tumour cell lines. The efficacy was lower in the case of the A375P line, while in the case of the SK-MEL-28 cell line, a strong response to the Ver-SNPs was observed. PDT with Ver-MSNs caused oxidative stress, activating HepG2 and finally leading to apoptosis [121,122]. Thus, Ver-MSNs showed a strong potential of selectivity against melanoma with a high degree of invasion, reducing the proliferation of cancer cells by half after minimal irradiation. The difference observed for the two cell lines is based on their different absorption, the endocytosis capacity being directly proportional to the invasiveness of the tumour (Table 2) [121,123].

**Figure 6:**
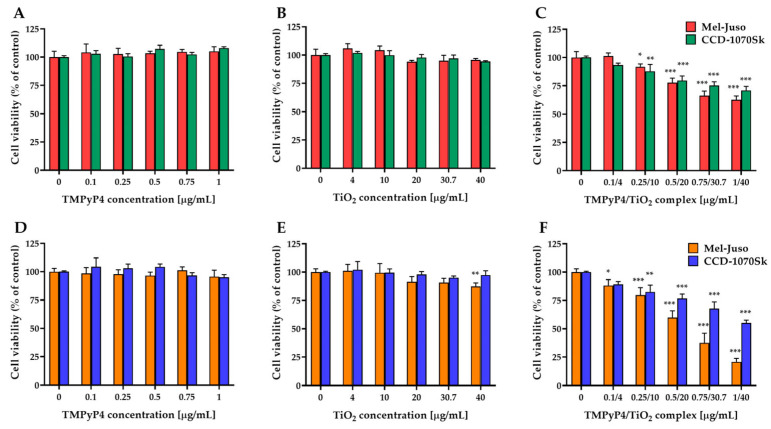
Effect of TMPyP4, TiO_2_ NPs, and TMPyP4/TiO_2_ complex on the metabolic activity/cell viability of cells. The graphs present the relative mitochondrial dehydrogenase activity of treated human Mel-Juso and CCD-1070Sk cells under dark (**A**–**C**) and light-irradiation conditions (**D**–**F**). Untreated cells (0 μg/mL) were used as control. Results (control vs. sample) were significant at *p* < 0.05 (*), *p* < 0.01 (**), and *p* < 0.001 (***). Error bars reflect the standard deviation. Reproduced from ref. [125].

In addition, refs. [124,125] investigated the effect of photoactivation of two porphyrins (5,10,15,20-(Tetra-4-sulfonate phenyl) porphyrin tetraammonium (TPPS) and 5,10,15,20-(Tetra-N-methyl-4-pyridyl) porphyrin tetra tosylate (TMPyP4)) encapsulated on γ-Fe_2_O_3_ NPs and, respectively, TiO_2_ NPs against an amelanotic MelJuso cell line. The results showed that TPPS-γ-Fe_2_O_3_ NPs conjugates destroyed the melanoma cells after only 1 min of exposure and that the TMPyP4-TiO_2_ NPs conjugates induced a phototoxicity of 78% under irradiation for 7.5 min with a 405 nm light and 1 mW/cm^2^ power density (Figure 6, reproduced from ref. [125]).

To improve palladium porphyrin (PdTCPP) delivery, Chen et al. investigated the efficiency of layered double hydroxides (LDH) as nanocarriers. The complex’s efficiency was tested in mice with skin-induced melanoma using B16-F10 cancer cells. In vivo, tests indicated that PDT performance reduced tumour growth sevenfold compared to the control mice group. The LDH-PdTCPP nanocomposites demonstrated low cytotoxicity, with no significant change in the mice’s body weights or fluctuations in relative organ weights, indicating that the nanocomposites were safe and could be a potential nanocarrier for PDT. The LDH nanocarrier promotes internalization into the cellular cytoplasm and raises the intracellular density of singlet oxygen. Also, LDH can serve as an in vivo contrast agent for oxygen sensing to image tumour hypoxia [126].

Ogawara et al. used PDT to develop a better and safer method of treating different types of cancer cells. They synthesized a block copolymer of polyethylene glycol and polylactic acid (PN-Por) nanoparticles functionalized with a hydrophobic porphyrin derivative, photoprotoporphyrin IX dimethyl ester (PppIX-DME) [127]. In vitro phototoxicity tests revealed that the nanocomplex has a substantial inhibitory effect on many types of cancer cells, including B16BL6 melanoma, and the efficacy was determined by the quantity of loaded PppIX-DME. In vivo, the polymeric nanoparticles were tested on C26 (Colon-26 carcinoma) tumour-bearing mice and presented a low accumulation in the liver and spleen, and consequently, PN-Por remained in the bloodstream for an extended period, which resulted in the effective targeting and accumulation of the drug in the tumour. Moreover, a substantial efficient tumour-fighting effect was observed in mice with C26 tumours. This occurred when local light doses of 150 W (halogen lamp with 600 nm cutoff filter) for 5 min were applied to the tumour tissues following the administration of PN-Por [127].

Plant-virus-based scaffolds are cost-effective and can be produced in large quantities. These protein-based nanoparticles are uniform in size and have well-defined atomic structures. Plant viruses are non-infectious to mammals but are biocompatible and biodegradable, making them suitable for medical applications in vivo. The nucleoprotein components of the tobacco mosaic virus (TMV) nanotubes were used to encapsulate the cationic porphyrin, 5-(4-ethynylphenyl)-10,15,20-tris-(4-methylpyridin-4-ium1-yl) porphyrin-zinc(II) triiodide (Zn-EpPor), for PDT. Zn-EpPor is a special cationic porphyrin with a zinc molecule in its ring, which enhances its accumulation in mitochondria and stabilizes its structure, improving its therapeutic effectiveness. This unique characteristic makes it a suitable candidate for melanoma PDT. The PDT delivery system’s cell binding and uptake were evaluated using flow cytometry and confocal microscopy. Zn-EpPorTMV showed a 40% increase in fluorescence uptake, up to 1347, compared to free Zn-EpPor, which was of 956 (*p* < 0.05%). According to the MTT cell viability assay, the Zn-EpPorTMV complex demonstrated significant anti-melanoma activity, with IC50 values of 0.54 µM and 0.24 µM for free Zn-EpPor and Zn-EpPorTMV, respectively, after 30 min of irradiation. The live/dead cell viability assay further confirmed the therapeutic efficacy at 5.0 µM concentration after 30 min of irradiation, while no cell death was observed in the dark controls. The Zn-EpPorTMV particle proved to be stable and efficacious in vitro, improving upon the cell targeting, uptake, and killing versus free Zn-EpPor. Based on the biocompatibility and tumour-homing properties of TMV, photosensitizer-TMV platforms such as Zn-EpPorTMV may hold promise for application in PDT or combination therapies targeting melanoma or other cancers. [128].

In summary, porphyrins are essential for the design of third-generation PSs, and the compounds investigated up to now for melanoma treatment are broadly synthesized in Table 2.

**Table 2:** Overview of compound types, NP types, irradiation parameters, cell lines used in the assays, and corresponding results.

Porphyrin Derivatives	NPs	Parameters	Cell Line	Results	References
5-ALA	Au	628 nm, 220 V, 50 Hz He–Ne laser, 20, 40, 60, and 80 J/cm^2^ doses	Mel-Rm	The best results were obtained when the 5ALA-gold NP conjugate was irradiated with an optical dose of 60 J/cm^2^	[113]
5-ALA	MFLs	532 nm laser 300 mW/cm^2^, for 5 min	B16-F10	The results indicate that at a 5-ALA concentration of 2 mmol/L, the complex after laser irradiation yielded to 20% cell viability.	[116]
PpIX (C_34_H_34_N_4_O_4_)	Poly (D, L lactic-co-glycolic acid) NPs (PLGA NPs)	Wavelength: 630 nm	B16-F10	PDT effect on melanoma was observed from a low PS concentration in nanocomplex (7.8 µg/mL).	[119]
Palladium-meso-tetra (4-carboxyphenyl) porphyrin (PdTCPP)	Layered double hydroxides	532 nm diode laser, 250 ± 5 mW cm^−2^	B16-F10	LDH-PdTCPP PS induces cytotoxicity against the B16-F10 melanoma cell line. PDT using the LDH-PdTCPP complex reduced tumour growth in mice sevenfold compared to the control group.	[126]
Photo protoporphyrin IX dimethyl ester (PppIX-DME).	Polyethylene glycol and polylactic acid (PN-Por)	Halogen light source, 15 s irradiation time in vitro, using light guide and cut-of wavelengths below 600 nm	B16-F10	The effectiveness of the nanoparticle complex is given by PDT irradiation. The complex of porphyrin derivative and polymeric NPs did not show tumour tissue specificity.	[127]
PpIX (C_34_H_34_N_4_O_4_)	Polysilsesquioxane (PSilQ) NPs	630 nm, 24.5 mW/cm^2^, 20 min	A375	The phototoxicity of the complex was nine times lower than the phototoxicity of porphyrin, thus reducing the side effects of NPs on normal tissue. PpIX-PSilQ NPs showed no cytotoxicity even at equivalent concentrations of PpIX as high as 250 µM.	[120]
Verteporfin (Ver)	Silica NPs (SNPs)	Wavelength: 650 nm	A375P and SK-MEL 28	After irradiation, proliferation of Ver-MSN-treated normal cells and inhibitory effects on tumour cell lines showed a lower metastatic effect in the case of the A375P line. After irradiation, SK-MEL 28 cell proliferation was reduced to half for Ver-MSN nanocomplexes, compared with the free Ver, which inhibits the cell growth by only 30%.	[121]
5,10,15,20-(Tetra-4-sulfonatophenyl) porphyrin tetraammo-nium (TPPS)	γ-Fe_2_O_3_ NPs (Iron Oxide NPs)	Wavelength: 405 nm,1 min blue light exposure, 1 mW/cm^2^	MelJuso	TPPS photodynamic activity had a significant increase via conjugation with γ-Fe_2_O_3_ NPs at a very low irradiation dose (1 mW/cm^2^ irradiation intensity and 1 min. of exposure) and with cytotoxicity at 1 μg/mL. Antitumour effect of γ-Fe_2_O_3_ NPs-TPPS for human melanoma cells subjected to PDT, through the generation of singlet oxygen. A 55% decrease in Mel-Juso cells treated with γ-Fe_2_O_3_ NPs-TPPS.	[124]
5,10,15,20-(Tetra-N-methyl-4-pyridyl) porphyrin tetratosylate (TMPyP4)	TiO_2_ NPs (Titanium Dioxide NPs)	405 nm LED, 1 mW/cm^2^, 7.5 min exposure time	MelJuso and CCD-1070Sk	The TMPyP4-TiO_2_ NPs conjugates enhance the porphyrin efficiency against human melanoma MelJuso cells while being less phototoxic on normal CCD-1070Sk skin fibroblasts, thus having a greater selectivity on cancer cells.	[125]
5-(4-ethynylphenyl)-10,15,20-tris-(4-methylpyridin-4-ium1-yl)porphyrin-zinc(II) triiodide (Zn-EpPor)	Nanotubes formed by nucleoprotein components of the tobacco mosaic virus (TMV)	White light from a Vivitek D950HD projector (~10 mW cm^−2^ at 430 nm) for 30 min	B16-F10	The Zn-EpPorTMV complex demonstrated significant anti-melanoma activity with a 40% increase in cellular uptake compared to Free Zn-EpPor and an IC50 value of 0.24 µM after 30 min of irradiation.	[128]

The reviewed studies summarised in Table 2 showed that porphyrin-based nanoplatforms such as porphyrin-based liposomes, micelles, and polymeric or metal oxides nanoparticles produce an enhancement in the PDT effect when compared with free porphyrin and resolve PDT limitations such as tumour selectivity, drug solubility, and cell internalization. As a versatile nanoplatform, the NPs loaded with porphyrin can also incorporate various imaging contrast agents enabling PET, fluorescence, MRI, CT, and photoacoustic imaging. Furthermore, the porphyrin-loaded NPs can also be used to incorporate other therapeutic agents like radionuclides, doxorubicin, paclitaxel, siRNA, and DNA, facilitating radiotherapy, chemotherapy, PTT, PDT, gene therapy, or a combination of therapies [129].

### 3.2. Chlorins

**Figure 7:**
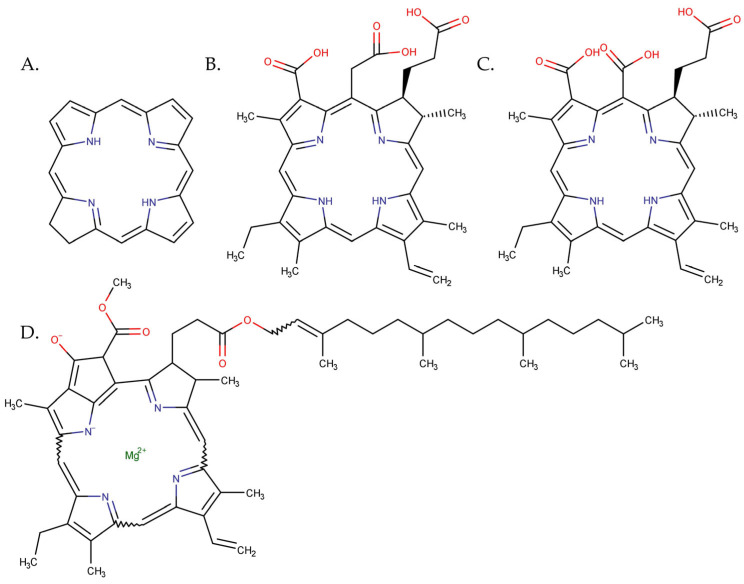
Two-dimensional structure of (**A**) chlorin; (**B**) chlorin e6; (**C**) chlorin p6; and (**D**) chlorophyll.

Chlorin pigments contain a reduced pyrrole ring in the tetrapyrrole structure compared to porphyrins (Figure 7) [130]. Chlorins are a second-generation PS with photosensitizing capabilities that make them a potential candidate for PDT in the treatment of melanoma. Light-activated chlorins produce ROS, which can cause oxidative damage and apoptosis, which leads to cell death [131,132].

There are already several clinically used chlorin-type PSs, namely, Foscan^®^, Bremachlorin^®^, Photodithazine^®^ (chlorin e6), and Laserphyrin^®^, which are used in the treatment of various types of cancer, including skin cancer [53,133].

To improve the solubility and stability and enhance its light absorption at longer wavelength, in red or even NIR range, several types of chlorin derivatives (chlorin e6, p6, meso-tetraphenyl chlorine disulfonate) were used in conjugation with NPs or other nanocarriers in PDT [134,135,136].

B16 mouse melanoma cell lines were used to determine the efficiency of chlorin derivatives conjugated with magnetic or super paramagnetic iron oxide NP. To improve tumour targeting, Mbakidi et al. used chlorin p6 (Figure 7C), a water-stable and -soluble chlorin that was conjugated to iron oxide NPs. For an enhanced permeability and retention effect, the magnetic NPs were grafted with dextran, a biopolymer that increases the plasmatic lifetime, and polyethyleneimine, an agent that improves the internalization of the nanocomplex in cancer cells. The antitumour activity was tested against two variants of the B16 mouse melanoma cell line (B16-F10 and B16-G4F, with or without melanin, respectively). The cell cultures were irradiated with an Aktilite lamp, an LED with emission centred at 630 nm [137]. The cell viability tests show that the complex has higher toxicity after LED irradiation compared with free PS. When the two variants of the melanoma cancer cells were evaluated, the results showed that higher photocytotoxicity was obtained on the B16-F10 cell line, indicating a possible specificity of the nanocomplex (Table 3) [131].

Chlorin e6 (Figure 7B) is a PS that is FDA-approved. This compound has high ROS generation and a 660 nm absorption peak [138,139]. Besides these favourable properties, the disadvantage of this PS is its hydrophobicity, which leads to the poor biodistribution and rapid clearance of the compound. To overcome these drawbacks, superparamagnetic iron oxide nanoparticles coated with polyglycerol were employed to deliver chlorin e6 and increase cell absorption. In addition, the chemotherapeutic agent doxorubicin was attached to the complex to increase affinity to the cell membrane and, hence, tumour cell uptake, resulting in a final delivery platform that consists of super magnetic iron oxide nanoparticles coated with polyglycerol and loaded with doxorubicin and chlorin e6. In this way, the chlorin e6 absorption in mouse melanoma cells was improved, resulting in enhanced photocytotoxicity defined by increased ROS formation, loss of viability, DNA damage, and promotion of tumour cell immunogenicity. Moreover, doxorubicin has a chemotherapeutic effect when released from the complex. The in vivo assays reveal the increase in the distribution and retention of chlorin e6 in mouse subcutaneous melanoma grafts and much better chlorin e6 PDT effect (Table 3) [140].

Table 3 provides a comprehensive synthesis of the chlorins that have been investigated thus far for the PDT of melanoma.

**Table 3:** Overview of compound types, NP types, irradiation parameters, cell lines used in experiments, and corresponding results.

Chlorins	NPs	Parameters	Cell Line	Results	Ref
Chlorin p6	Iron oxide	630 nm 37 J/cm^2^ fluence	B16-F10 B16-G4F	The highest phototoxic effect was obtained on B16-F10 cell line after irradiation indicated a possible specificity of the nanocomplex.	[131]
Chlorin e6	Superparamagnetic iron oxide	690 nm laser irradiation 0.5 W/cm^2^, 30 s	B16-F10	Chlorin e6 and doxorubicin conjugated with coated polyglycerol NPs have increased absorption in melanoma cells and enhanced photocytotoxicity.	[140]
Chlorin e6	Liposomes	In vitro: 660 nm continuous laser (50 mW/cm^2^, 5 min). In vivo: 660 nm laser (200 mW/cm^2^, 10 min)	A375	PGIL synergistically achieves a high-efficiency PDT effect by enhancing apoptosis, inhibiting invasion, and boosting NK cell-related immune effects in melanoma cells.	[141]
Chlorin e6	Polymer DSPE–PEG2000–biotin	In vitro 2D: ultrasound radiation 1 MHz, 50% duty cycle, 1.5 W/cm^2^, 2 min and/or LED irradiation 405 nm, 0.5 J/cm^2^; In vitro 3D: ultrasound radiation 1 MHz, 50% duty cycle, 1.5 W/cm^2^, 2 min and/or two-photon laser excitation at 730 nm, 15 mW, 5 min; In vivo: ultrasound radiation 1 MHz, 50% duty cycle, 1.5 W/cm^2^, 2 min 730 nm, 35 mW, 5 min	A375	In vitro and in vivo tests show that synergistic action of the ultrasonic and light irradiation of the complex lead to enhanced cytotoxic effect and almost fully eradicate the melanoma tumour in the mouse model.	[132]
Chlorin e6	Aluminium-albumin	In vitro: 660 nm laser irradiation, 0.8 W/cm^2^, 5 min In vivo: 660 nm laser irradiation, 0.8 W/cm^2^, 5 min, single dose in day 0	B16-F10	Al-BSA-Ce6 NPs inhibited growth of the first tumour, significantly prolonged survival, also reduced possibility of recurrence, and inhibited growth of tumour cells in distal and lung metastases by stimulating specific tumour immune response.	[142]
Chlorophyll	Pluronic F68 nanocomposite	In vitro: 671-nm laser irradiation, 20 min In vivo: 671 nm laser, 20 min, at 24 h, for 15 days	A375	The photothermal and PDT effect of encapsulated vegetable chlorophyll into Pluronic F68 polymeric micelle proved high efficiency against melanoma in vitro and in vivo compared with non-encapsulated.	[143]
Ferrous chlorophyllin	Liposomes	monochromatic red laser 652 nm, 200 mW/cm^2^ 56.2 J/cm^2^	B16-F10	The cellular uptake of liposomes increased over time (6 to 24 h) via endocytosis, with preferential accumulation in the mitochondria and nucleus; after depigmentation, PDT with liposomes containing Fe-CHL resulted in an LC50 value of 1.77 μM after 48 h incubation, causing cell death through a combination of apoptosis and necrosis.	[41]

On the A375 human melanoma cell line, Chen et al. investigated the efficacy of chlorin e6 (Figure 7B) conjugated with cyclometalated iridium (III) and encapsulated in an amphiphilic polymer DSPE-PEG 2000-biotin NP. The complex is able to localize in mitochondria and can be excited via ultrasonic radiation and two-photon laser irradiation to treat deeply invasive tumours. The studies reveal that the complex is collected in the mitochondria after an 8 h incubation, which is the perfect target for producing a therapeutic effect. Two-dimensional monolayer cells and a three-dimensional multicellular spheroid model were tested in vitro. The cells were exposed to ultrasonic radiation and/or 405 nm LED irradiation in the case of the 2D model, and the results reveal the cell death induced by complex’s synergistic action following ultrasonic and light irradiation. The A375 3D multicellular spheroid model was also exposed to ultrasonic radiation and/or two-photon light irradiation at 12 h after incubation. The data showed that the use of both sonodynamic and two-photon dynamic therapy led to significant cancer remission. The two-photon light absorption demonstrated luminescence up to 180 μm, showing that two-photon excitation can be exploited for deep tissue penetration. In vivo studies were performed on A375 tumour-bearing mice that were subjected to ultrasonic radiation and/or two-photon laser irradiation 12 h after the treatment injection. The results demonstrate that when the treatment is combined, the tumour size is dramatically decreased and is almost eliminated during a single session (Table 3) [132].

Liposomes loaded with chlorin e6 and low-molecular-weight citrus pectin were used to create a photoactivable Galectin-3-inhibitor nanoliposome (PGIL). PGIL was designed to improve PDT and activate immune cells called NK cells to fight melanoma. Galectin-3 (Gal-3) is a protein involved in cancer cell proliferation, apoptosis, and metastasis and represents a promising target for cancer therapy. Low-molecular-weight citrus pectin (LCP) is a natural compound that contains galactoside structures that inhibit tumour growth and metastasis, induce tumour cell apoptosis, and activate antitumor immune responses by blocking Gal-3 function. The cellular uptake, inhibitory activity, cytotoxicity, and PDT activity of PGIL were assessed in A375 melanoma cells and fibroblasts. The results showed that PGIL was able to accumulate in tumour cells and inhibit their growth. It also enhanced the PDT effect of chlorin e6, when exposed to 660 nm diode continuous laser (50 mW/cm^2^, 5 min) light. After PDT treatment, PGIL showed enhanced cell killing and inhibited tumour cell invasion. Additionally, PGIL improved NK cell-related immune response. The combined effects of PDT and immune activation make PGIL a promising strategy for melanoma treatment, as studied in A375 cells and tumour-bearing nude mice. These results suggest that PGIL could be a promising new treatment for melanoma. It has the potential to kill cancer cells directly, inhibit their growth, and activate the immune system to fight cancer [141].

Zhu et al. developed chlorin e6 (Ce6)-containing albumin (BSA) NPs that may be photoactivated to destroy tumour cells. They also integrated aluminium in the form of aluminium hydroxide into NPs to boost the immune system and to reduce the potential of immunological escape of tumoral cells. The tumour recurrence or metastasis might be thus avoided by PDT using Al-BSA-Ce6 NPs [142]. In vitro results show that after NIR irradiation (5 min at 660 nm, 0.8 W/cm^2^) of treated B16-F10 cell cultures, Al-BSA-Ce6 generated many more ROS than free Ce6. Also, irradiating Al-BSA-Ce6 0.5 μg/mL at a sufficiently low power (0.15 W/cm^2^) to avoid photothermal cytotoxicity reduced cell viability by 89.8% compared to only 36.8% for free Ce6. NIR laser irradiation for 5 min of Al-BSA-Ce6 NPs at a concentration of only 0.1 μg/mL killed 94.68% of the cells compared to only 10.83% in the case of free Ce6. NP-encapsulated Ce6 was taken up through endocytosis mediated by clathrin, caveolae, and cholesterol, as well as by micropinocytosis. In vivo tumour targeting by Al-BSA-Ce6 NPs and their antitumor effect were also investigated. After intravenously injecting B16-F10 tumour-bearing C57BL/6 mice with Al-BSA-Ce6 or free Ce6 (5 mg/kg), the research team collected the hearts, livers, lungs, spleens, kidneys and tumours at 3, 6, 9, 12, and 24 h after injection. The ex vivo fluorescence imaging analysis showed that at 9 h after injection, Al-BSA-Ce6 NPs showed a nearly 4-fold greater accumulation in tumours than free Ce6. This accumulation ensures ROS generation specifically in the tumour region. Mice bearing B16-F10 tumours were intravenously injected with Al-BSA-Ce6 NPs at 5 mg/kg and irradiated at 660 nm for 5 min on day 0. Some of these mice were also injected subcutaneously with TLR9 agonist CpG around the tumour. By day 15, tumours in most mice treated with Al-BSA-Ce6 NPs had shrunk to become nearly undetectable. Histology of tumour tissue at 9 days after irradiation showed that Al-BSA-Ce6 NPs led to the largest area of tumour cell killing, without causing substantial toxicity to other organs besides moderate inflammation. Moreover, 62.5% of mice treated with Al-BSA-Ce6 NPs and CpG survived for 100 days, compared to only 37.5% of mice treated with Al-BSA-Ce6 NPs alone (Table 3) [142].

On the cell line A375, Chu et al. [143] applied vegetable chlorophyll (Figure 7D) encapsulated into Pluronic F68 polymeric micelles to improve the water solubility. The vegetable-extracted chlorophyll has the advantage of non-toxicity, of having a near-infrared-laser-induced thermal effect, and of having fluorescence properties that can be used for imaging. The nanocomposites were investigated for tumour target imaging and synergetic photothermal and photodynamic effect after 671 nm laser irradiation. According to the study findings, Pluronic F68 chlorophyll nanocomposites were able to target melanoma cells and mouse tumours. The nanocomposite generated high levels of intracellular ROS after 20 min irradiation. The viability quantitative test showed that after the incubation of A 375 cells with the nanocomposite followed by 20 min irradiation, a cell viability of 0.36% at the highest chlorophyll concentration of 0.5 mg/mL was contained in the complex, whereas without irradiation, the cell viability stayed close to 100%. Furthermore, without Pluronic encapsulation, chlorophyll alone killed only 5% of the cells with the same irradiation dose. In vivo, the Pluronic F68 chlorophyll nanocomposites were able to target mouse tumours. After 20 min of laser irradiation, every 24 h for 15 days, the nanocomposites demonstrated tumour eradication. The study’s in vitro and in vivo results showed that the nanocomposite’s synergistic photothermal and photodynamic activities attained a high anti-melanoma efficacy of dietary chlorophyll [143].

Chlorophyll derivatives like chlorophyllin–metal complexes with (M = Fe, Mg or Cu) have several advantages, including good water solubility and excellent photosensitivity at long wavelengths that can penetrate tissues well. Among these derivatives, ferrous chlorophyllin (Fe-CHL) displays the strongest PDT activity [41]. Liposomes, which are biocompatible nanocarriers, can encapsulate and stabilize photosensitizers either in their lipid bilayer or aqueous core and improve skin penetration, PS localization, and circulation time. The study used an MTT assay to determine cell viability, TEM analysis to examine cellular uptake and localization of liposomes, and flow cytometry to evaluate apoptotic and necrotic melanoma cells after PDT. The TEM investigation of melanoma cells shows that after 6 h, most liposomes were located on the cell’s outer surface; after 12 h, liposomes were observed in the cytoplasm, nucleoplasm, and nuclear chromatin; and at 24 h, maximum liposome density was observed in the mitochondria, nucleoplasm, and nuclear chromatin, with less accumulation in the cytoplasm. No significant cellular toxicity was found in dark toxicity experiments using the MTT assay. However, liposomes-encapsulated Fe-CHL-mediated PDT induced a significant decrease in cell viability. The LC_50_ values were determined to be 18.20 µM at 24 h and 1.77 µM at 48 h [41].

TEM examination of melanoma cells was conducted on depigmented cells (Phenylthiourea has been used as a melanin synthesis inhibitor that causes partial depigmentation of melanoma cells) treated with the LC_50_ conditions of liposomes-encapsulated Fe-CHL followed by PDT with a light dose of 56.2 J/cm^2^ revealed the presence of necrotic, apoptotic, and late apoptotic mechanisms in melanoma cell death. This study shows the potential success of Fe-CHL-mediated PDT for treating melanoma using liposomal delivery systems [41].

In summary, these results show the significance of nanocarriers based delivery methods for improving the therapeutic effectiveness of chlorins derivatives as PS in melanoma PDT treatment. In all of the reviewed studies, the use of the nanocarrier for PS delivery has anti-melanoma efficacy at lower concentrations for the same irradiation dose when compared with the chlorins alone. The suitable wavelength range for irradiation proved to be between 671 and 730 nm, and those wavelengths are associated with a good tissue penetration around 4 to 5 mm (Table 3) [144]. To confirm these results and investigate the use of these nanosystems in therapeutic settings, further studies have to be developed.

### 3.3. Phthalocyanines

Phthalocyanines (Pc), a second-generation PS, are aromatic heterocycles composed of four isoindole rings linked by nitrogen atoms [54]. Phthalocyanines have two primary electronic absorption bands—the Soret band (between 300 and 400 nm) and the Q band (between 600 and 700 nm) [145].

**Figure 8:**
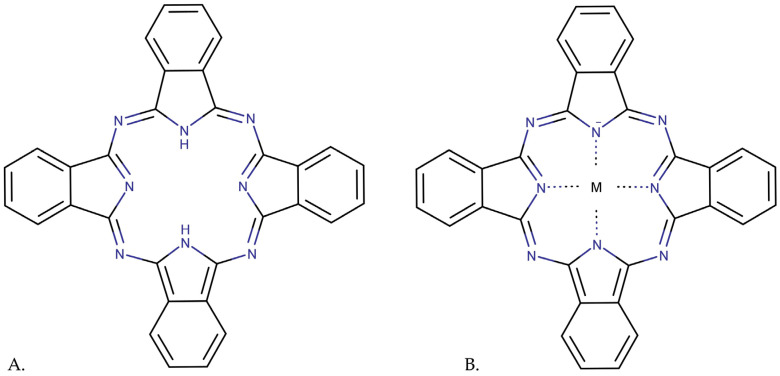
Structural formulae of (**A**) non-metallated and (**B**) metallated phthalocyanines. Adaptation from Staicu et al. [146].

The two primary types of phthalocyanine compounds are non-metallated phthalocyanine and metallated phthalocyanine. In the centre of metallated phthalocyanines is a metal atom. Cu, Ni, Fe, Al, Zn, Au, Ag, Co, Mn, and Mg are metals found in metallated phthalocyanines [146]. In comparison to metal-free analogues, phthalocyanine complexes with diamagnetic ions may be promising photosensitizers. Aluminium complexes (AlPc, ClAlPc), zinc complexes (ZnPc, ZnPcS_2_P_2_-photocyanine), and silicon complexes (SiPc4) are well-known examples of metallated phthalocyanines. The coordination of a central metal ion within phthalocyanines (Figure 8) significantly favours the intersystem crossing from the singlet to triplet state, increasing triplet lifetime and leading to higher singlet oxygen generation quantum yield [54].

In various studies testing the efficiency of diverse phthalocyanine PSs in complex with nanoparticles, B16 melanoma cell lines were employed as an experimental model. In their study, Bolfarini et al. [147] examined the impact of zinc phthalocyanine (ZnPc) conjugated with cucurbituril, wherein cucurbituril was employed as a means to enhance the aqueous solubility of the ZnPc compound. An ultra-stable magnetic fluid based on citrate-coated cobalt ferrite nanoparticles was used as a nanocarrier for the cucurbituril ZnPc conjugate. Following the thin lipid film preparation method, a cationic magnetoliposome containing both magnetic fluid and the photosensitizer-based complex was obtained. The therapeutic synergism between PDT and magnetohyperthermia was assessed through experimental evaluation of the magnetoliposomes on the B16-F10 melanoma cell line. The colorimetric MTT assay with or without light and magnetic field treatments was used to assess cell viability after PDT and magnetohyperthermia. The results reveal that cell viability after administration of the magnetoliposomes is not affected for control cells without PDT or magnetohyperthermia. Although the magnetoliposomes activated by PDT are more effective than in the case of magnetohyperthermia, the data indicate that the combination of the two methods is significantly more effective than any treatment separately delivered (Table 4) [147].

Do Reis et al. [148] introduced a dual-encapsulated polymeric NP system as a promising alternative treatment approach for melanoma. The nanosystem was prepared by initially mixing ZnPc with polylactic acid (PLA), followed by the addition of the chemotherapeutic agent dacarbazine, and ultimately combining it with polyvinyl alcohol (PVA). The ZnPc, owing to its additional charge resulting from the incorporation of zinc, exhibits enhanced interactions with negatively charged membranes, thereby facilitating improved skin penetration. Furthermore, the presence of the chemotherapeutic agent dacarbazine promotes acidification of the NP system, thereby enhancing permeation, and it may facilitate targeted therapy against the MV3 melanoma cell line.

To assess its efficacy, the MV3 human metastatic melanoma cell line was subjected to three variants of the nanosystem: empty PLA/PVA NPs, PLA/PVA NPs encapsulating dacarbazine, and PLA/PVA NPs encapsulating dacarbazine and ZnPc.

The in vitro PDT tests demonstrated that the encapsulation of both dacarbazine and ZnPc is essential to have an increased efficacy, and it is dose-dependent. The study was conducted by pre-incubating the cells with each nanosystem for 24 h and 72 h before the laser irradiation. If the PDT effect is the same for 24 h and 72 h for a higher dose of 100 µg, in the case of 20 µg, the results revealed a higher cell inhibition after 72 h compared to 24 h, suggesting a potentially slower internalization and drug release process.

The toxicity test conducted on endothelial cells showed that the dacarbazine contained within nanoparticles did not show an effect when compared to the control samples. On the other hand, the in vivo results revealed that the amount of the drug loaded into the nanoparticles affected how they were distributed throughout the body. The low accumulation of these nanoparticles in the stomach, heart, brain, and kidneys indicated a potential reduction in the typical side effects associated with Dacarbazine.

The in vitro cell toxicity assay using endothelial cells demonstrated that the dacarbazine encapsulated into nanoparticles had no significant toxicity compared to control samples. In vivo results demonstrated that drug loading affects the biodistribution of the nanoparticle formulations. The low accumulation of the NPs into the stomach, heart, brain, and kidneys suggested that common side effects of dacarbazine could be reduced. This innovative approach, incorporating both PDT and chemotherapy within a single NP system, presents a promising and novel avenue for melanoma treatment (Table 4) [148].

The synthesis and application of gold nanoparticles (AuNPs) stabilised by the co-self-assembly of the hydrophobic ZnPc and a water-soluble thiol-functionalised poly (ethylene glycol) (PEG) (ZnPc- AuNPs -PEG) for the in vivo delivery and PDT of amelanotic melanoma was reported by Camerin et al. [149]. C57/BL6 mice having a subcutaneously transplanted B78H1 amelanotic melanoma were injected with the ZnPc-AuNPs-PEG conjugates. The pharmacokinetic studies revealed that the conjugates’ retention times in the serum and the tumour were enhanced in comparison to nanoparticles functionalized with ZnPc alone. The bile–gut pathway allowed the conjugates to be removed without significant toxicity. The irradiation of the tumour-bearing mice was performed at 3 h, 24 h, and 1 week after intravenous injection of the ZnPc-AuNPs-PEG conjugate using a halogen lamp emitting in the 620–700 nm wavelength range isolated by optical filtering. The light source was operated at a fluence rate of 175 mW/cm^2^ for a total fluence of 157 J/cm^2^ for 15 min. The mice that were irradiated at 3 h after receiving the ZnPc-AuNPs-PEG conjugates had the best response to PDT. All of the mice in this group lived for 18 days after PDT, and 40% were totally cured, as evidenced by a lack of tumour development up to 45 days. It is considered that the tumour response to PDT consists of primarily vascular damage [149].

The in vivo antitumoral activity of Zn(II)-phthalocyanine disulphide (C11Pc), a compound with both phthalocyanine units containing seven hexyl chains and a sulphur terminated C11 chain, was examined for C57/BL6 mice with subcutaneously implanted amelanotic melanoma (B78H1 cell line). The study assessed the antitumoral activity as well as the pharmacokinetics of free C11Pc and C11Pc conjugated to gold NPs. The pharmacokinetic investigations revealed that 3 h post-injection, a higher amount of C11Pc-NP is detectable in the serum compared with free PS, which accumulates faster in the spleen, liver, and lung. At 24 h after injection, the C11Pc-NP demonstrated the highest accumulation within the neoplastic lesion. As a result, PDT studies were conducted at these two specific post-injection time points. The C11Pc or the C11Pc-NP samples in a Cremophor emulsion was administered via the caudal vein, approximately 10–15 days after subcutaneous injection of the melanoma cell line. The C11Pc dose used was 1.5 μM/kg body weight. The PDT irradiation source was a halogen lamp filtered to emit in the 600–700 nm range with a total fluence of 157 J/cm^2^. The result shows that PDT performed at 3 h after injection of the C11Pc-NPs demonstrated a significantly more extensive tumour response compared with the free compound. The study observed the photodamage of the vascular system of the tumour and an optimal slowing effect of tumour growth following light treatment 3 h after injection. Although the delivery by nanoparticles increased the efficiency of PDT, further studies are needed to overcome the persistence of the nanocomplexes in important organs such as the liver and spleen (Table 4) [150].

**Table 4:** Overview of compound types, NP types, irradiation parameters, cell lines used in experiments, and corresponding results.

Phthalocyanine Derivatives	NPs	Parameters	Cell Line	Results	Ref
Zinc phthalocyanine (ZnPc)	Magnetic fluid, containing citrate-coated maghemite NPs	Magnetohyperthermia assay: AC magnetic field operating at 1 MHz and 40 Oe amplitude, for 3 min PDT assay: 670 nm, laser diode, 600 mW average power, at 84 mW/cm^2^ light irradiance, light dose between 0.5 and 2 J/cm^2^	B16-F10	The combined application of laser light and AC magnetic field on B16-F10 cells incubated with the magnetoliposome formulation resulted in a significant reduction in cell viability compared with PDT or magnetic field alone.	[147]
ZnPc	Polylactic acid (PLA)/ polyvinyl alcohol (PVA)	660 nm laser, irradiance of 28 J/cm^2^ for 2.5 min	MV3	PLA/PVA-encapsulated dacarbazine and ZnPc substantially augmented cell death in MV3 cells following PDT.	[148]
ZnPc	Gold-PEG conjugates	Halogen lamp (620–700) nm, fluence rate of 175 mW/cm^2^, total fluence of 157 J/cm^2^, 15 min	B78H1	Irradiation of the amelanotic melanoma at 3 h following i.v. injection of the ZnPc-AuNPs-PEG conjugates induced a photodynamic destruction of the tumour. In addition, 40% of the mice were completely cured, with no tumour regrowth.	[149]
Aluminium chloride phthalocyanine (ClAlPc)	SLN	670 nm, diode Eagle laser, average power of 0.30 mW and a light radiance of 17 mW/cm^2^; 0.5, 1.0, and 2.0 J/cm^2^ light doses	B16-F10	The best results were obtained when the ClAlPc-SLN complex was used at the highest dosages: 2.0 J/cm^2.^with a cell viability of 15.06%.	[151]
ClAlPc	NLC and SLN	630 nm LED, total fluence of 25.3 J/cm^2^	BF16-F10	ClAlPc-free exhibited 100% cell viability regardless of LED irradiation, while NLC 40 at concentration of 0.2 μg/mL decreased the cell viability to 0.93%. Without irradiation, NLC 40 caused a significant reduction in cell viability, with 12% at a concentration of 0.2 μg/mL of the drug.	[152]
Aluminium phthalocyanine (AlPc)	SLN	660 nm LED, 10 min, at 10 cm distance, with 25.88 J/cm^2^ fluence	B16-F10	After LED exposure, SLN-AlPc demonstrated a decrease in cell viability, demonstrating the potential of PDT for the targeted killing of cancer cells. Higher PDT activity observed with SLN-AlPc-20μM.	[153]
Zn(II)-phthalocyanine disulphide (C11Pc)	Gold	600–700 nm wavelength range form quartz-halogen lamp; at a fluence-rate of 175 mW/cm^2^ for a total fluence of 157 J/cm^2^	B78H1 transplanted in C57 mice	PDT studies show that tumour growth is slowed following light activation of C11Pc conjugated to gold NP.	[150]

An exhaustive list of the phthalocyanine derivatives that have been developed and studied so far for melanoma PDT is provided in Table 4.

Aluminium chloride phthalocyanine (ClAlPc) can boost photodynamic activity on melanoma due to its optical absorption range between 600 and 800 nm. Similar to the most phthalocyanines, the major drawback is its hydrophobicity. Solid lipid nanoparticles (SLN) were used to overcome the constraints of hydrophobicity by promoting controlled PS release, lower toxicity, increased stability and bioavailability, and the delivery of ClAlPc in monomeric form. In addition, compared to other colloidal carriers, SLNs have the advantages of not needing organic solvents, cost-effectiveness, and large-scale manufacturing possibilities [151]. The ClAlPc-SLN nanocomplex was produced using the direct emulsification process. Its phototoxicity was evaluated in vitro on the B16-F10 cell line. The study had four groups: cells treated with 0.75 g/mL ClAlPc-loaded SLNs, cells treated with free ClAlPc, a negative control group, and a laser control group. Laser irradiation was applied to all groups, using light doses of 0.5, 1.0, and 2.0 J/cm^2^ at a wavelength of 670 nm. Twenty-four hours after laser irradiation, the cell viability was assessed using the MTT assay.

The findings of this study demonstrate a clear correlation between the efficacy of the ClAlPc-SLN nanocomplex and the dose of irradiation, as evidenced by the MTT assay results. Specifically, the encapsulated ClAlPc exhibited a significantly lower cell viability of 15.06% at a light dose of 2 J/cm^2^, compared to 54.12% at 0.5 J/cm^2^. In contrast, the free ClAlPc compound displayed higher cell viability of 48.9% at an irradiation dose of 2 J/cm^2^, highlighting the higher efficacy of the nanocomplex compared to the free form of ClAlPc. These results provide valuable insights into the enhanced therapeutic potential of the ClAlPc-SLN nanocomplex for targeted photodynamic therapy (Table 4) [151].

Almeida et al. [152] also investigated the antitumour effect of ClAlPc on the BF16-F10 cell line. To enhance both the antitumour effect and skin penetration, they employed both types of lipid nanoparticles (LNs), SLNs and nanostructured lipid carriers (NLC). The LNs were prepared using stearic acid as the solid lipid and oleic acid as the liquid lipid, with a ratio of 20% oleic acid (NLC 20) or 40% oleic acid (NLC 40). To assess skin penetration and permeation, in vitro experiments were conducted using pig ear skin as a model, and in vivo studies were carried out on hairless mice.

The stratum corneum, the skin’s primary barrier, is effectively penetrated by the LNs, according to both in vitro and in vivo data. Only 10.5% of the NLC 40 formulation was kept in the stratum corneum, compared to the control formulation’s approximately 73% retention rate. In the NLC 40 formulation, 89.5% penetrated the skin and reached the deeper layers. This improved permeation of NLC40 can be attributed to the occlusive effect brought on by the topical application of LNs, which increases skin hydration while improving the drug penetration capacity. In addition, this can be due to the LNs’ nanometric size, which makes it easier for them to transport the PS into the deeper layers of the skin. The efficacy of the PDT in melanoma using ClAlPc-free and NLC 40 (with PS concentrations ranging from 0.0125 μg/mL to 0.2 μg/mL) was evaluated using the MTT assay. For the irradiation protocol, a 630 nm LED was used at a total fluence of 25.3 J/cm^2^. After irradiation on melanoma BF16-F10, an average cell viability of 100% was observed for ClAlPc-free. NLC 40 with concentrations of 0.012, 0.025, and 0.05 μg/mL of ClAlPc did not reveal significant antitumour effects, while at 0.1 and 0.2 μg/mL, cell viability was significantly reduced to 36% (*p* < 0.001) and to 0.93%, respectively. The study concludes that the presence of OA in the LNs seems to enhance the antitumour effect and that NLC may be favourable for ClAlPc encapsulation in the PDT of melanoma [152].

**Figure 9:**
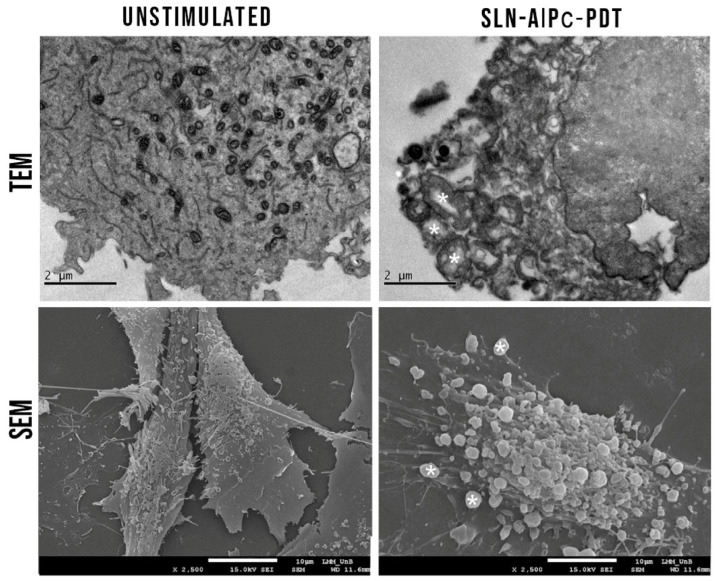
B16-F10 cells morphology in TEM and SEM after the PDT treatment with SLN-AlPc; it was also possible to observe formation of apoptotic bodies (*) and cytoplasmic bumps [153].

Mello and colleagues conducted studies to investigate the efficacy of an Amazon butter-based SLN loaded with aluminium phthalocyanine (AlPc) on the B16-F10 melanoma cell line [153]. For the MTT assay, two concentrations of AlPc, 20 μM and 40 μM, were used for the SLN-AlPc complexes. The cells were irradiated using a 660 nm wavelength LED for 10 min, with a total dose of 25.88 J/cm^2^. The results showed a significant decrease in cell viability after irradiation. The IC_50_ values were found to be 19.62 nM for SLN-AlPc-20 μM and 53.84 nM for SLN-AlPc-40 μM. The higher PDT activity observed with SLN-AlPc-20 μM compared to SLN-AlPc-40 μM might be attributed to the formation of AlPc aggregates at higher concentrations [153]. Regarding B16-F10 cell morphology under PDT, the formation of apoptotic bodies and cytoplasmic bumps was observed (Figure 9, reproduced after ref. [153]). The authors concluded that PDT can promote apoptosis through the activation of caspases and decrease the expression of apoptosis-inhibiting proteins, such as Bcl-2.

In summary, phthalocyanines offer several advantages as PSs, including good light absorption at longer wavelengths and a high quantum yield in ROS formation. Their hydrophobic nature and tendency for aggregation, however, limit their usefulness. To address these issues, several nanocarriers and modifications to the phthalocyanine structure has to be developed. Such nanoplatforms as Zinc phthalocyanines encapsulated in PLA/PVA or magnetic fluid containing citrate-coated maghemite NP inhibit MV3 or B16-F10 melanoma cell lines. Lipid nanoparticles such as SLN or NLC proved to be effective carriers for hydrophobic phthalocyanine ClAlPc. These lipid-based nanocarriers increase ClAlPc’s bioavailability, stability, and controlled release, resulting in improved therapeutic effects. These nanocomplexes have shown higher photodynamic effectiveness, solubility, and photo-stability, resulting in significant cell damage in melanoma cell lines.

## 4. Non-Porphyrin Photosensitizers

**Figure 10:**
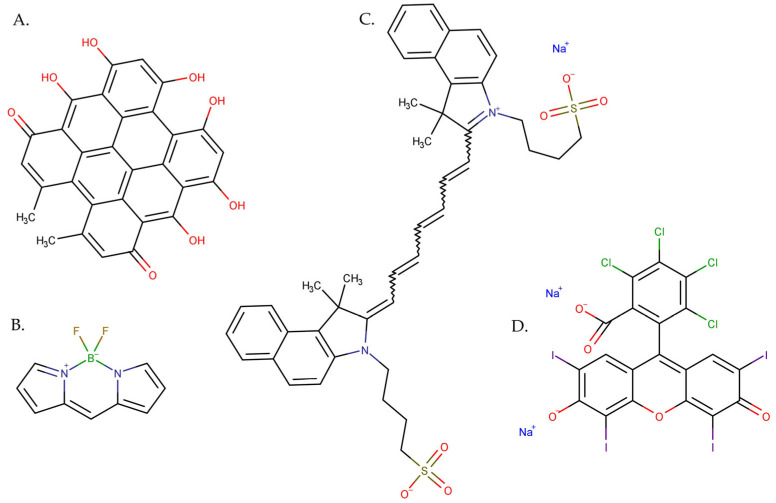
Two-dimensional structure of non-porphyrin photosensitizers: (**A**) hypericin, (**B**) BODIPY, (**C**) indocyanine green, and (**D**) Rose Bengal.

Non-porphyrin-based PSs belong to the second generation of PSs. When compared with porphyrin-based PSs, their application in cancer treatment is considerably behind. Squaraines, cyanines, xanthenes, anthraquinones, phenothiazines, curcuminoids, or boron–dipyrromethene (BODIPY) are examples of dyes or natural compounds used in cancer PDT [154]. Some of these chemical structures are shown in Figure 10.

Some synthetic dyes (halogenated cyanine dyes synthesized by introducing chlorine, bromine, and iodine into the structure of heptamethine dye) help to obtain a synergistic effect between PDT and photothermal therapy (PTT), leading to enhanced cancer cell death [155].

Cyanine dyes are compounds that absorb light in the visible to near-infrared-I (NIR-I) spectrum range (750–900 nm). These dyes offer a wide range of derivatives due to various structural modifications, such as halogenation, the incorporation of metal atoms or organic structures, and the synthesis of lactosomes, emulsions, or conjugation. These modifications are aimed to increase solubility in aqueous media, enhance phototoxicity, and reduce photobleaching. Cyanine and its derivatives have shown potential as photosensitizers, capable of efficient response to light activation and leading to the death of target cells through apoptosis. To enhance specificity for cancer tissues, cyanine dyes can be sensitized to pH, thereby increasing their phototoxicity in an acidic environment, which is characteristic for extracellular cancer fluid [74].

Indocyanine green (ICG) was initially used to measure cardiac output, avoiding the impact of fluctuations in blood oxygen levels. ICG has FDA permission for ocular angiography, measuring cardiac output, and evaluating liver blood flow and hepatic function [156,157]. ICG (Figure 10C), a water-soluble tricarbocyanine dye (maximum absorption at 800 nm) is a promising option for PDT because it produces singlet oxygen in the presence of light irradiation [158]. However, ICG has several drawbacks, including a short circulation half-life, concentration-dependent aggregation, and rapid excretion from the body. Encapsulating ICG with NPs offers a promising approach to address these drawbacks and overcome the associated limitations. Using nanocarriers to deliver ICG may be an attractive option for offering both diagnostic and therapeutic interventions in cancer treatment [81].

Tang et al. studied the synergistic effect of chemotherapy with PDT and PTT for melanoma treatment [159]. They encapsulated temozolomide (TMZ) and ICG in the NPs of modified carboxylated poly(amido-amine) (PAMAM). The artificial dendrimer PAMAM was treated with succinic anhydride (SA) in order to modify the positive surface charge, changing most of the amino groups into carboxyl groups, thus obtaining PAMAM-COOH. Later, the formed NPs were coated with hyaluronic acid (HA) to allow active targeting of the tumour cells, as well as to enhance the stability and the encapsulation capability of the NPs [159,160].

The integration of ICG, along with the chemotherapeutic agent TMZ, in HA-altered PAMAM-COOH NPs led to better results in an anti-melanoma treatment than each compound tested separately. This suggests that part of the A375 cells were killed by the PTT/PDT effect from the ICG loaded in NPs when exposed to NIR (808 nm) radiation, and at the same time, the generated heat releases TMZ, which kills the remaining cancer cells. This synergistic activity induced almost total tumour ablation (Table 5) [159].

Another study of ICG was performed by Campu et al., who functionalized anisotropic gold diamond-like nano-bipyramids (AuBPs) with ICG in order to assess their PDT/PTT activity against murine melanoma B16-F10 cells [161]. Similar to ICG, AuBPs are also NIR-photoactivatable materials. ICG had the role of increasing the innate photodynamic and photothermal abilities of AuBPs, and it was shown that the created nanosystem generated a double quantity of singlet oxygen and raised the temperature by 2 °C, in comparison to the AuBPs alone. Prior to ICG loading, AuBPs were coated with polylactic acid (PLA), which improves the biocompatibility of the nanosystem and serves as a hydrophobic substrate for the anchoring of ICG (Table 5) [161,162]. The nanocomplex is also conjugated with folic acid (FA) that targets the folate receptor overproduced in the membranes of B16-F10 tumour cells [161]. A hybrid nanosystem consisting of PLA-coated AuBPs functionalized with ICG and further conjugated with FA (AuBPs@PLA@ICG@FA) was applied for 24 h on a melanoma cell culture, which was furthermore exposed for 15 min to 785 nm laser radiation. The combined PDT/PTT activity of the hybrid nanosystem led to more than 90% of the cancer cells being killed [161].

Promising results of ICG were obtained by Wen et al., who loaded the NIR dye into a bovine serum albumin–manganese dioxide complex (MnO_2_@BSA), thus synthetizing hydrogen-peroxide-responsive protein biomimetic nanoparticles (MnO_2_-ICG@BSA) [163]. The PDT/PTT effect of the MnO_2_-ICG@BSA nanoparticles was investigated on B16-F10 melanoma cells. Moreover, the in vivo effect was assessed on melanoma cells injected in nude mice. The PDT/PTT effect resulted in an approximately 35% survival rate of the melanoma cells after treatment with a concentration of 250 µg/mL of MnO_2_-ICG@BSA and 5 min laser irradiation and an approximately 25% survival rate after applying 500 µg/mL of the nanocomplex and 5 min laser exposure. The in vivo experiments showed that all mice treated with MnO_2_-ICG@BSA and exposed to NIR laser radiation survived, unlike the mice in the control group that died after 10 days [163].

The encouraging prospects of ICG for PDT in melanoma were also studied in [164]. This research presents a new formulation of a potential delivery system for ICG for topical applications. Lee et al. selected liposomes as the ICG’s nanocarriers, mainly because of their amphiphilic nature that would stabilize ICG molecules known to have a lipophilic character, as well as a hydrophilic one [164,165]. Another reason is that liposomes have the ability to permeate the skin, and utilizing them as carriers might improve ICG’s transdermal delivery [164,166]. The drawback to be considered in the loading of ICG into liposomes is that both are negatively charged. This could prevent ICG’s integration, and it may hinder the permeation of the new formulation through the skin. To overcome this impediment, the liposomes were coated with chitosan, a natural positively charged polysaccharide, with efficient uses in transdermal delivery [167]. ICG-loaded liposomes coated with chitosan had dimensions between 1000 nm and 2000 nm, depending on chitosan concentration [164]. Despite other studies finding that bigger liposomes fail to permeate the skin, chitosan-coated liposomes have the capacity to circumvent the size constraint in transdermal permeability by employing a variety of chitosan processes that increase penetration [168]. Similar results were reported in [164], where a skin permeation study showed that a larger amount of ICG was collected after 12 h from the chitosan-coated liposomes versus the uncoated ones. This study also shows the phototoxic activity of ICG. B16-F10 melanoma cells were treated with a concentration of 40 μM of ICG and exposed for 2.5 min to laser radiation at a 775 nm wavelength and 250 mW power. ICG-loaded liposomes coated with chitosan showed dark- and photo-cytotoxicity that were dependent on chitosan concentration. At higher concentration of chitosan (0.1%), the ICG-loaded liposomes’ cytotoxicity was enhanced by exposure to radiation. These results demonstrated the potential of ICG-loaded liposomes coated with chitosan in PDT on melanoma cells (Table 5) [164].

**Figure 11:**
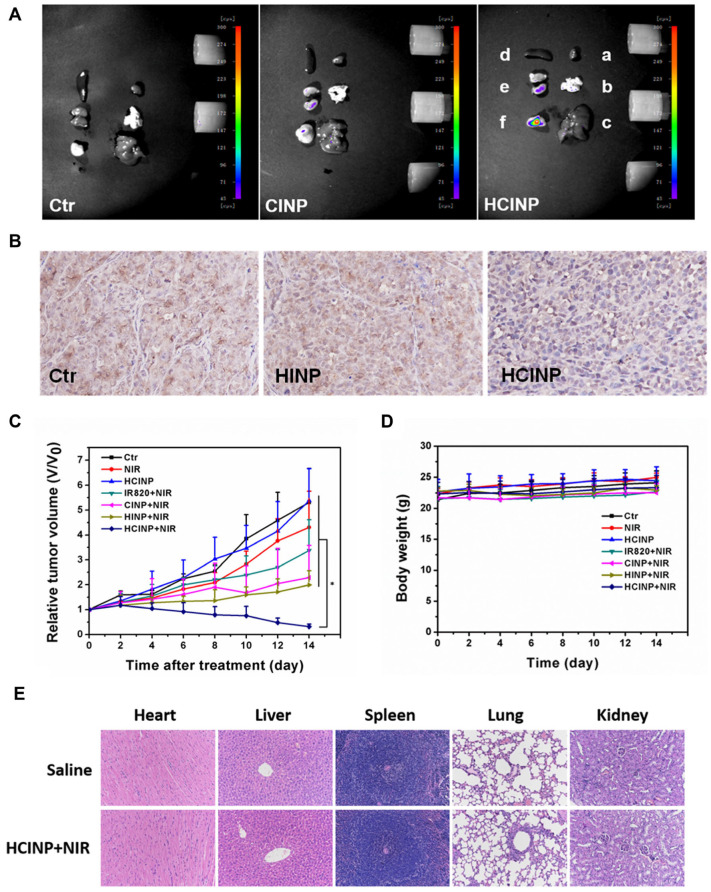
Enhanced antitumour effect of HCINPs. (**A**) Measurement of the in vivo targeting effect of HCINPs (a: heart, b: lung, c: liver, d: spleen, e: kidney, f: tumour). (**B**) Expression of HIF-1α in tumour tissues (200×). The mean positive area of the control group, HINP group, and HCINP group were 1522, 1503, and 1092, respectively. (**C**) Relative tumour volume curves of different groups of MV3 tumour-bearing mice. * *p* < 0.05, versus the HCINP group. (**D**) Body weights of mice measured during the 14 observation days in different groups. (**E**) HE-staining images of major organs collected from the saline (Normal) and HCINPs with NIR irradiation groups (200×). Reproduced from ref. [170].

A cyanine dye, IR820, having asimilar chemical structure to ICG, is a promising candidate for PDT, PTT, and fluorescence imaging, but with better in vitro and in vivo stability, which may be attributed to the addition of a chlorinated cyclohexene as an intermediate ring [169]. In their study, Hou et al. [170] incorporated IR820 and catalase (CAT) into poly(lactic-co-glycolic acid) (PLGA) NPs, and to improve their ability to target tumour cells, hyaluronic acid (HA) was modified on the surface of the nanoparticles to construct HA-PLGA-CAT-IR820 nanoparticles (HCINPs). The authors report a new approach to relieve tumour hypoxia and to improve IR820-based PDT against malignant melanoma. The HCINP formulation was applied on human melanoma cell lines MV3, M14, and A375, and human skin fibroblast cells, HSFs. The cell cultures were treated with 8 μg/mL drug formulations for 12 h, and afterwards, they were exposed for 5 min to NIR laser radiation emitted at 808 nm with the power density of 4 W/cm^2^. In vitro experimental results showed that in the HCINP + NIR group, almost all cells in the irradiated area died, showing a strong tumour cell killing effect. For in vivo tests, following drugs injections, the mice were irradiated in the same conditions as for in vitro trials. After 14 days, all mice were sacrificed, and their main organs (heart, liver, spleen, lung, and kidney) were dissected for pathological biopsy. In the targeting analysis of HCINPs in vivo, experimental results proved that this formulation can specifically accumulate in the tumour and cause insignificant effects on other tissues. The authors found that the tumours almost completely disappeared by the 14th day in the HCINPs + NIR group, revealing an enhanced PDT effect to effectively kill melanoma cells (Figure 11 [170]). The assessment of the therapeutic safety of HCINPs + NIR in vivo performed by measuring the mice body weights during treatment, as well as via histopathological examination of major organs, proves that no significant physical or pathological changes occurred after 14 days of HCINPs + NIR treatment compared with that in the untreated group. The results showed that the novel nanoplatform HCINPs could selectively target melanoma cells with high expression of CD44, and generated oxygen by catalysing H_2_O_2_, which increased the amount of singlet oxygen, significantly inhibiting tumour growth in the end (Table 5) [170].

Similar to ICG, Rose Bengal (4,5,6,7-tetrachloro-20,40,50,70-tetraiodofluorescein disodium—RB) is a synthetic dye with an amphiphilic character. Part of the fluorescein class, this anionic xanthene dye is medically approved for diagnosing corneal injuries. RB has intrinsic tumour and bacterial cytotoxicity, but it is FDA-approved only as an orphan medicine for the treatment of specific malignancies. It is a photosensitizer characterized by a high singlet oxygen quantum yield, which makes it appropriate for PDT. Although RB offers therapeutic promise, its limitations (e.g., short half-life, low intracellular uptake) imply that it should be delivered through nanocarriers [82,83].

An example of nanocarriers utilized for the delivery of RB are organically modified mesoporous silica nanoparticles (MSNs). MSNs are biocompatible silica systems having a large surface area (800 m^2^/g), as well as large and uniform pore volume (0.9 cm^3^/g), which permit significant drug intake [171,172]. In order to load RB on MSNs, these were functionalized with amine groups (NH_2_-MSNs). RB links to the amine groups through covalent bonds, leading to the formation of RB-MSNs. The RB-MSNs’ quantum yield of singlet oxygen generation evaluated under exposure to 540 nm radiation was 0.74, almost the same as the one of free RB. The PDT effect of RB-MSNs was investigated on an aggressive melanoma cellular model (SK-MEL-28). Cell proliferation was reduced after the melanoma cells were incubated with RB-MSNs for 5 h and then exposed for 5 min to green light [171]. Bazylińska et al. developed a double core nanoplatform to deliver RB together with trioctylphosphine oxide (TOPO)-stabilized NaYF_4_: nanoparticles co-doped with Er^3+^ (2%) and Yb^3+^ (20%) (NaYF_4_:Er^3+^,Yb^3+^) [173]. RB and the NPs were co-encapsulated in spherical polymeric nanocarriers formed from poly(lactide-co-glycolide) (PLGA) copolymer, stabilized by Span 80 and Cremophor A25 (non-ionic surfactants). The in vitro activity was assessed on wild-type human melanoma granular fibroblasts (MeWo), a cell line originating from lymph node metastasis of skin melanoma (Me-45) and, as a control, a human cutaneous keratinocyte line (HaCaT). The melanoma and control cells were incubated for 24 h with the loaded double core nanoplatform. After incubation, they were exposed for 5 min at a radiation from a lamp (HOP 250, OPTEL, Poland) with wavelengths between 520 and 560 nm and fluence rate of 10 J/cm^2^. After irradiation, the cells were further incubated, and the photocytotoxicity was evaluated after 24 h and 48 h. A cell viability substantially reduction was observed for both cancer cell lines after exposure to green light, the best result being obtained for MeWo cells (cell viability diminished with more than 90%). Still, the effect of RB-loaded nanocarriers was lower than the one of free RB on control cell line, indicating that the double core nanoplatform protects healthy cells during PDT. Additionally, due to the good results obtained, MeWo cells were selected for more tests. These were incubated for 4 h with the nanoplatforms loaded with RB and NaYF_4_:Er^3+^ and Yb^3+^ and then were irradiated for 5 min with a laser diode emitting at a 980 nm wavelength and with an intensity of 6.2 W/cm^2^. Afterwards, the melanoma cells were incubated overnight with a monoclonal mouse F-actin antibody in order to determine if the reactive oxygen species affected the cells’ normal activity. The findings suggested that PDT induced damages and restructuration of the F-actin fibres inside the cytoskeleton. In conclusion, the study reports successful PS delivery into cancer cells, as well as significant PDT efficacy enhanced by NIR-activation of the encapsulated hybrid cargo in skin melanoma cells [173].

RB derivatives with enhanced amphiphilicity were developed by Chen et al. for sono-photodynamic therapy [174]. To create these novel derivatives, RB was conjugated with methoxypolyethylene glycols, known for their ability to enhance biocompatibility and facilitate cellular uptake. Alternatively, RB was also linked with quinoline fragments, which are recognized for their extensive biological activity, including potent antitumoural effects. One of the RB derivatives formed by coupling with a methoxypolyethylene glycol fragment proved to be more hydrophilic in comparison with the other ester. This observation suggests that the more hydrophilic derivative likely possesses an extended chain length, which could potentially promote the self-assembly to hydrophilic nanoparticles. The photodynamic, sonodynamic, and sono-photodynamic anticancer effects of the RB derivatives were assessed on different cancer cell lines (human hepatocellular carcinoma HepG2 cells, breast cancer cell line MCF-7, and murine melanoma cell line B16-F10). It was suggested that RB conjugation with an appropriate methoxypolyethylene glycol fragment can lead to a combined and more potent anticancer effect by enhancing intracellular ROS generation and by boosting cellular uptake [174].

Part of the phenothiazine class, methylene blue (MB) is a dye and a medicine, as well as having photosensitizing activity. Mohseni et al. evaluated the photodynamic effect of MB loaded in hollow gold nanoshells (HGNSs) on human breast cancer MCF-7 and DFW melanoma cancer cell lines [175]. Prior to MB loading, HGNSs were coated with polyethylene glycol (PEG) in order to improve the NPs’ half-life, to facilitate bypassing the immune system, and to limit unwanted medicine release. HGNSs-PEG-MB were tested at three different concentrations (5 µM, 10 µM, 20 µM), and their PDT effect was assessed at 24 h after exposure to radiation at 670 nm, with an intensity of 14.9 mW/cm^2^, for 3, 6, and 9 min. The effect was dependent on concentration and exposure time, and for both cell lines, the best results were obtained for an HGNSs-PEG-MB concentration of 20 µM and 9 min exposure time, decreasing the cell survival rate to 2% for DFW and at 3% for MCF-7. This study showed that HGNSs-PEG improved MB intracellular uptake and enhanced the PDT effect compared to MB alone [175].

Another study that assesses the effectiveness of PDT using MB-loaded NPs is mentioned in [176]. However, in contrast to the previously presented study, [176] examines not only the effect of MB alone but also its combined effect with veliparib, which is a poly(adenosine diphosphate (ADP)-ribose) polymerase (PARP) inhibitor. The PARP damage-repair signalling pathway might be involved in the PDT resistance mechanism; thus, inhibiting PARP might improve the PDT effect. MB and veliparib were co-encapsulated in PLGA NP, and the in vitro effect of VMB-NPs was evaluated. B16-F10-Nex2 melanoma cells were incubated with VMB-NPs containing different concentrations of MB and veliparib and exposed to radiation (660 nm, 102 J/cm^2^). A decrease of 36% in cell viability was observed after PDT treatment for the concentrations of 1 µM of MB and 8.3 µM of veliparib. The study suggested that MB kills the cells via ROS generation during PDT, while veliparib inhibits the regeneration of photodamaged melanoma cells; thus, their synergistic activity improves treatment efficacy [176].

Quinzarin dyes, belonging to the quinone family, exhibit various pharmacological activities, but their use is limited by their poor solubility in aqueous medium, leading to inefficient biodistribution. To overcome this, incorporating quinizarin into a poly(methyl methacrylate) nanostructured system can enhance its effectiveness and targeted delivery at the desired site. MTT cytotoxicity assays on the B16-F10 melanoma cell line revealed the toxicity of PP-NP at 268 μg/mL and QZ-PP-NP at concentrations ranging from 167 to 222 μg/mL in the dark (without PDT). PDT assays using blue LEDs were conducted on the melanocytic cell line B16-F10 with two concentrations of PP-NP or QZ-PP-NP, 111 and 222 μg/mL, and at fluences ranging from 1.0 to 25 J/cm^2^. An increase in QZ-PP-NP concentration and LED light fluence resulted in significantly greater induction of cell death compared to the control. Flow cytometry analysis indicated that photodynamic treatment with QZ-PP-NP and LED light triggered apoptotic cell death, with up to 60% of cells in the apoptotic pathway. The necrotic population remained below 15%, consistent with results obtained with other photosensitizers. Confocal microscopy assays showed that in melanocytic cells, QZ was distributed in the whole cell, with a higher concentration in the nucleus [177].

An overview of the non-porphyrin PSs that have been synthesized and explored up to now for the PDT of melanoma is provided in Table 5.

**Table 5:** Overview of compound types, NP types, irradiation parameters, cell lines used in experiments, and corresponding results.

Non-Porphyrin PS	NPs	Parameters	Cell Line	Results	Ref
ICG	HA-PAMAM-COOH	808 nm, 1.5 W/cm^2^, 10 min	A375	Almost total tumour ablation due to synergistic effect of PDT, PTT, and chemotherapy after treatment with ICG- and TMZ-loaded HA-PAMAM-COOH exposed to NIR radiation.	[159]
ICG	AuBPs	785 nm, 190 mW, 15 min	B16-F10	More than 90% of the melanoma cells were killed due to the combined PDT/PTT effect after treatment with hybrid nanosystem AuBPs@PLA@ICG@FA.	[161]
ICG	MnO2@BSA	808 nm, 1 W/cm^2^, 5 min	B16-F10	A 65–75% kill rate of the melanoma cells, according to used MnO_2_-ICG@BSA concentrations, due to PDT/PTT effect.	[163]
ICG	Chitosan-coated liposomes	775 nm, 250 mW, 2.5 min	B16-F10	Cytotoxicity was enhanced by chitosan concentration. At 0.1% chitosan concentration, ICG-loaded liposomes’ cytotoxicity was increased by irradiation (approximately 70% cell viability).	[164]
IR820	CAT-PLGA-HA	808 nm, power density of 4 W/cm^2^, 5 min	MV3, M14, and A375	The novel drug delivery nanoplatform could be used to alleviate hypoxia in the tumour microenvironment and improve the efficacy of PDT, providing a foundation for further research into novel melanoma treatment techniques.	[170]
RB	NH_2_-MSNs	540 nm, 5 min	SK-MEL-28	Reduced cell proliferation after irradiation.	[171]
RB + NaYF_4_:Er^3+^,Yb^3+^	PLGA	520–560 nm, 10 J/cm^2^, 5 min	MeWo and Me-45	Significant reduction in cell viability for both melanoma cell lines (>90% for MeWo).	[173]
RB + NaYF_4_:Er^3+^,Yb^3+^	PLGA	980 nm, 6.2 W/cm^2^, 5 min	MeWo	Restructuration and destabilization of cytoskeletons F-actin fibres.	[173]
RB conjugated with methoxypolyethylene glycol	Self-assembly hydrophilic NP	λ > 500 nm, 27 J/cm^2^, 30 min	HepG2, MCF-7 and B16-F10	Boosted cellular uptake, enhanced intracellular ROS generation, improved synergistic anticancer efficacy.	[174]
MB	HGNSs-PEG	670 nm, 14.9 mW/cm^2^, 9 min	DFW and MCF-7	A 2% cell survival rate for DFW and a 3% cell survival rate for MCF-7, at a HGNSs-PEG-MB concentration of 20µM.	[175]
MB + veliparib	PLGA	660 nm, 102 J/cm^2^	B16-F10-Nex2	Cell viability decreased by 36% for 1 µM concentration of MB and 8.3 µM concentration of veliparib.	[176]
Quinizarin	Poly-(methyl methacrylate)	blue LED 450 (±20 nm), fluences ranging from 1.0 to 25 J.cm^−2^	B16-F10	PDT assays demonstrated significantly increased cell death with higher QZ-PP-NP concentration and LED light fluence, mainly inducing apoptotic cell death.	[177]
I2-BDP	UiO-66	visible light irradiation at a power density of 80 mW cm^−2^ for 10 min	B16-F10	The IC_50_ for the light-activated UiO-PDT on B16-F10 cell line was obtained at a concentration of 0.70, μg mL^−1.^	[178]
I_2_BDP	PCN-222	405 nm, 4 mW/cm^2^, 3 h	B16-F10	Irradiation of PCN–I_2_BDP(Nano) with light induces high cytotoxic activity.	[179]
Hyp	F127-FA/ F127-SN/ F127-BT	550−625 nm, 35 mW/cm^2^, 46.8 J/cm^2^ light dose, 40 min	B16-F10	CC_50_ of 0.24 ± 0.02 µmol/L for Hyp—F127-BT	[180]
Curcumin	Silica	blue LED 465 nm; 34 mW/cm^2^	A375	Curcumin–Si nanocomplex in PDT displays low toxicity on normal cells, but toxic effect on A375 cells at 50 µg/mL, inducing apoptosis, inhibiting cancer cell proliferation, and enhancing intracellular ROS generation.	[181]
ISQ	BSA-AuNC@AuNR	808 nm, power density of 1 W/cm^2^, 4 min	A375	The combined effect of the targeted photo- and chemotherapies produced an appreciable toxicity to the melanoma cells.	[182]

A family of non-porphyrin PSs in the PDT of melanoma have emerged from the 4,4-difluoro-4-bora-3a,4a-diaza-s-indacenes (also known as boron–dipyrromethene, BODIPY), a class of fluorescent dyes with an increasing number of applications including medicine, pharmacology, and environmental sciences. BODIPY’s structure usually contains a BF_2_ bridging unit, and the addition of heavy halogen atoms, such as Br and I, in the pyrrole rings increases the triplet yield and provides them with excellent photosensitizing abilities [183]. BODIPY is photochemically stable and has good absorption properties in the NIR spectral range. Furthermore, recent research has investigated the integration of metals into the BODIPY core, providing further options for exploration and improvement of its properties [154]. BODIPY derivatives and their NP complexes have gained significant attention owing to such properties as ease of structural synthesis, high photostability, rapid clearing in normal tissues, and good photo–dark toxicity ratio [184].

Wang et al. incorporated carboxyl-functionalized diiodo-substituted BODIPYs (I2-BDP) into zirconium-based nanoscale metal–organic frameworks (UiO-66), resulting in the final product UiO-PDT. UiO-PDT and I2-BDP were tested with or without light activation at different concentrations, scaling from 0.1 to 6.25 µg/mL on the B16-F10 cell line using an MTT assay. For irradiation protocol, the authors used visible light at a power density of 80 mW/cm^2^ for 10 min. The results indicate that in the absence of irradiation, both I2-BDP and UiO-PDT present low toxicity, while light-activated compounds show high inhibition in a dose-dependent manner. The UiO-PDT demonstrated strong cellular uptake over time, effective singlet oxygen production, and high biocompatibility, making it a suitable option for photodynamic therapy in cancer treatment [178].

The design, synthesis, and biological activity of a novel BODIPY-incorporated metal–organic framework (MOF) were reported by Oh et al. [179]. The all-in-one PS complex incorporates an iodine-substituted BODIPY (I_2_BDP) into the porphyrin nanoscale metal–organic frameworks (NMOF), PCN-222 that consists of [Zr_6_(μ_3_-OH)^8^(–OH)^8^]^8+^ nodes, in which eight of the twelve octahedral edges are coordinated to 5,10,15,20-tetrakis(4-carboxyphenyl)porphyrin (TCPP) ligands. The new PCN–I_2_BDP nanocomplex with its rigid structure, prevents the self-quenching of the PS and exhibits high anticancer efficacy in PDT attributed to synergism between the BODIPY and the porphyrin molecules. The anticancer efficacy of PCN–I_2_BDP was in vitro tested on B16-F10 melanoma cells after 3 h LED light exposure (405 nm) at a 4 mW/cm^2^ irradiation dose. The authors reported an IC_50_ value of 9 nM against B16-F10 mouse melanoma and an improvement in the activity in the light phase of 10,000 times in contrast to the dark activity. Moreover, the authors observed that nanoparticles enter the cancer cells and spread in the cytosol.

The naturally occurring compound hypericin (Hyp), which has a long history of usage in traditional medicine, is found in *Hypericum perforatum* L. Recent studies have revealed its multifunctional qualities and possible therapeutic uses. Although further research is required to validate these findings, and the processes are not fully understood, studies show that it has anti-depressive, antineoplastic, anticancer, and antiviral properties. Hyp has become a potential treatment for cancer therapy and detection through photodynamic activation [185].

Hyp’s hydrophobicity restricts its physiological solubility and causes self-aggregation and diminished phototherapeutic effectiveness. Nanocarriers are used to improve the solubility, stability, and transport of the Hyp as a PS. Through the increased permeability retention effect, these systems offer passive targeting, permitting the regulated and steady release of the PS. This method allows for precise biodistribution management and increases PS accumulation at the therapeutic target site [180]. For improved Hyp delivery and targeted melanoma cell treatment, De Morais et al. produced multifunctional systems employing the Pluronic F127 copolymer covalently joined with biotin (BT), F127-BT, spermine (SN), F127-SN and folic acid (FA), and F127-FA to increase the therapeutic PDT efficacy on melanoma. FA, a soluble B-complex vitamin, improves tumour targeting, boosting treatment selectivity and effectiveness while minimising possible side effects to healthy cells. BT exhibits promise as a nanomaterial ligand, with specificity for receptors found on cancer cells. As a result, the coupling of BT with copolymers can improve nanomaterial absorption, facilitating targeted distribution and increasing therapeutic efficacy. SN is a cancer biomarker that can improve the interaction between the drug and the tumour target and enhance drug uptake, selectivity, and efficacy. The study investigates the potential of these drug delivery systems as a theragnostic platform for the treatment of melanoma. The efficacy of Hyp-loaded multifunctional micelles as a PS was assessed in vitro on B16-F10 melanoma cells. The results showed improved Hyp absorption by B16-F10 cells due to the conjugation of binders (FA, BT, SN) with the Pluronic F127, which enhanced PDT. These findings confirm the potency of multifunctional nanocarriers as a targeted therapy approach for the management of melanoma. The nanomicelles-incorporated Hyp showed a 50% cytotoxic concentration CC_50_ under illumination of 0.47 ± 0.02 µmol/L for Hyp—F127-FA; 0.77 ± 0.03 µmol/L for Hyp—F127-SN; 0.24 ± 0.02 µmol/L for Hyp—F127-BT; and 1.28 ± 0.11 µmol/L for Hyp- F127. The best results were thus obtained when the Pluronic F127 copolymer was covalently joined with SN and loaded with Hyp [180].

Ghazaeian et al. explore the potential of curcumin–silica nanocomplexes as a photosensitizer in PDT for melanoma cancer treatment using human melanoma cancer cells (A375). Curcumin, a natural polyphenol compound, has demonstrated antioxidant, anti-inflammatory, and anticancer properties. However, its low solubility in water limits its application as an effective photosensitizer. To overcome this limitation, curcumin was loaded onto silica-based NPs, which improved its solubility and enabled controlled release under unconventional pH and temperature conditions. The nanocomplex’s interaction with haemoglobin and double-stranded DNA shows no adverse effects on the biological function of haemoglobin and successful binding to DNA. Cell toxicity experiments were performed on human melanoma cancer cells (A375) and human fibroblast cells as normal cells using different concentrations of curcumin and curcumin–Si nanocomplex (0,10, 25, 50, and 100 mg/mL). PDT experiments were conducted with a blue LED (465 nm; power density: 34 mW/cm^2^). The results revealed that the nanocomplex exhibited significantly higher PDT effects on A375 cell death when compared to free curcumin or normal cells with or without PDT. When the antioxidant ascorbate was administrated before PDT, it demonstrated a protective effect on A375 cells, further confirming the involvement of ROS in inducing cell death. Fluorescence microscopy and flow cytometry analyses were employed to investigate the cellular effects of the nanocomplex PDT. The study revealed that the PDT with the curcumin–silica nanocomplex induced notable morphological changes in melanoma cancer cells and significantly increased ROS generation, leading to apoptosis and necrosis. The findings offer a new viewpoint on using PDT in cancer therapy, while further studies are needed to reveal the mechanism of action [181].

Recently, a novel melanoma-targeted theragnostic nanoenvelope (MTTNe) has been built by assembling a bovine serum albumin (BSA)-stabilized gold nanocluster on a gold nanorod (BSA-AuNC@AuNR) for a three-in-one combined therapy (PTT, PDT, and chemotherapy), enhanced with diagnosis via surface-enhanced Raman scattering (SERS) detection technique. The resultant MTTNe was coloaded with the melanoma-specific, FDA-approved drug dacarbazine (DAC) and a newly synthesized NIR absorbing squaraine molecule, ISQ, that served both as a photosensitizer and multiplex Raman sensor. In order to target highly expressed cell death receptors specific to melanoma, anti-DR5 monoclonal antibodies were attached to the nanoplatform. Upon 808 nm (power density of 1 W/cm^2^, 4 min irradiation time) single laser trigger, significant photoeffects of MTTNe were initiated, resulting in photothermal hyperthermia and ^1^O_2_-driven photodynamic effect in the presence of ISQ, followed by on-demand thermoresponsive drug release in the intracellular environment. Raman imaging was used to track the drug release kinetics and target-specific identification on melanoma cells using a multiplex SERS spectral pattern of ISQ (1345 cm^−1^) and DAC (1269 cm^−1^). The effectiveness of the MTTNe as a therapeutic agent was assessed using in vitro cytotoxicity tests in human melanoma cells (A375), and the apoptotic phenomenon was confirmed through molecular-level observation of intracellular SERS signatures. Protein denaturation and DNA fragmentation were the major cellular processes during apoptosis which were reflected in the Raman spectral bands of specific functional bonds. Finally, in vivo subacute toxicity tests on BALB/c mice were performed to evaluate the biocompatibility of MTTNe. The ex vivo haematoxylin and eosin staining suggested that BSA-AuNC@AuNR is mainly harmless in nature, although MTTNe showed very minimal toxicity to liver, kidney, spleen, heart, and lungs [182].

Finally, non-porphyrin PSs, such as ICG, RB, Hyp, squaraines, and BODIPY, have shown significant potential in PDT. The use of non-porphyrin PSs in conjugation with nanocarriers and/or other therapeutic agents is encouraging for enhancing PDT’s efficacy in melanoma therapy. These advancements and their combined effects with additional therapies may represent a promising strategy for improved cancer treatment.

## 5. Conclusions and Future Perspectives

PDT has emerged as a promising treatment strategy for melanoma. Traditional delivery systems often face challenges associated with poor solubility, limited stability, and rapid clearance from the body, hindering their therapeutic efficacy.

Nanoplatforms have garnered significant interest as potential carriers for PSs in PDT due to their unique properties. Various nanoplatforms, such as metallic, polymeric, or solid lipid nanoparticles; liposomes; micelles; and metal–organic frameworks, possess remarkable capabilities for encapsulating diverse bioactive compounds, including drugs and imaging agents, and may increase photosensitizer solubility, extend circulation times, enhance drug delivery, improve tumour penetration, enhance tumour accumulation, and provide controlled release. Nanocarriers allow the controlled release of a PS, leading to a more effective generation of ROS within the tumour cells, thereby increasing the therapeutic effect. Moreover, nanoplatforms exhibit enhanced photostability, making PDT more efficient. Additionally, nanoparticles can be engineered to have multifunctional capabilities, enabling them to carry out various therapeutic functions simultaneously, further improving the efficacy of PDT.

However, while nanoparticles hold great promise, they also present challenges that need to be addressed including their potential toxicity or tendency to form agglomerates that may hinder their proper function in targeted therapy. The biocompatibility of specific nanoparticles may be an issue, which warrants close examination to ensure safe application in clinical settings. Furthermore, the extended clearance time and the risk of accumulation raise concerns. High production costs and scalability issues may hinder their widespread use and accessibility in clinical settings. Regulatory challenges regarding safety, efficacy, and long-term effects on patients and tumour heterogeneity are also important aspects that need careful consideration. Liposomes are biodegradable and biocompatible and can encapsulate polar and non-polar bioactive agents, providing sustained release and targeted delivery. However, their drawbacks include relatively low stability and a tendency to agglomerate. Polymeric nanoparticles offer excellent stability, controlled synthesis, and tailored drug release patterns, making them attractive for various therapeutic purposes. Nevertheless, they face challenges, including agglomeration and the potential for nanotoxicity associated with non-biodegradable polymers, necessitating thorough evaluation and modification for safe and effective application.

Nanocarriers have numerous advantages for photodynamic therapy, and their successful integration into clinical practice depends on addressing biocompatibility, toxicity, and regulatory considerations. Further research and well-designed clinical trials are indispensable to validating their efficacy and safety.

Overall, this study provides a thorough overview of the current state of the art in PDT for melanoma and also highlights the immense potential and prospects for future advancements. By encouraging further research in this area, the findings synthesized in this review open new perspectives for developing more efficient and targeted treatments, leading to improved patient outcomes and enhancing their quality of life. As researchers continue to build upon these foundations, it is hopeful that this cutting-edge approach will eventually become a mainstream and highly effective therapeutic strategy in the fight against melanoma and other forms of cancer.

## Data Availability

Not applicable.

## References

[1] Arnold M., Singh D., Laversanne M., Vignat J., Vaccarella S., Meheus F., Cust A.E., De Vries E., Whiteman D.C., Bray F. (2022). Global Burden of Cutaneous Melanoma in 2020 and Projections to 2040. JAMA Dermatol..

[2] Radiation: Ultraviolet (UV) Radiation and Skin Cancer. https://www.who.int/news-room/questions-and-answers/item/radiation-ultraviolet-(uv)-radiation-and-skin-cancer.

[3] Zeng L., Gowda B.H.J., Ahmed M.G., Abourehab M.A.S., Chen Z.-S., Zhang C., Li J., Kesharwani P. (2023). Advancements in Nanoparticle-Based Treatment Approaches for Skin Cancer Therapy. Mol. Cancer.

[4] Wagstaff W., Mwamba R.N., Grullon K., Armstrong M., Zhao P., Hendren-Santiago B., Qin K.H., Li A.J., Hu D.A., Youssef A. (2022). Melanoma: Molecular Genetics, Metastasis, Targeted Therapies, Immunotherapies, and Therapeutic Resistance. Genes Dis..

[5] Vicente A.L.S.A., Novoloaca A., Cahais V., Awada Z., Cuenin C., Spitz N., Carvalho A.L., Evangelista A.F., Crovador C.S., Reis R.M. (2022). Cutaneous and Acral Melanoma Cross-OMICs Reveals Prognostic Cancer Drivers Associated with Pathobiology and Ultraviolet Exposure. Nat. Commun..

[6] Pillaiyar T., Manickam M., Namasivayam V. (2017). Skin Whitening Agents: Medicinal Chemistry Perspective of Tyrosinase Inhibitors. J. Enzym. Inhib. Med. Chem..

[7] Neagu M., Caruntu C., Constantin C., Boda D., Zurac S., Spandidos D.A., Tsatsakis A.M. (2016). Chemically Induced Skin Carcinogenesis: Updates in Experimental Models (Review). Oncol. Rep..

[8] Risks and Causes of Skin Cancer. https://www.cancerresearchuk.org/about-cancer/skin-cancer/risks-causes.

[9] Griffith C.F. (2022). Skin Cancer in Immunosuppressed Patients. JAAPA.

[10] Wang L.L., Lin S.K., Stull C.M., Shin T.M., Higgins H.W., Giordano C.N., McMurray S.L., Etzkorn J.R., Miller C.J., Walker J.L. (2023). Cutaneous Oncology in the Immunosuppressed. Dermatol. Clin..

[11] Zeng H., Li J., Hou K., Wu Y., Chen H., Ning Z. (2022). Melanoma and Nanotechnology-Based Treatment. Front. Oncol..

[12] Garbe C., Peris K., Hauschild A., Saiag P., Middleton M., Bastholt L., Grob J.-J., Malvehy J., Newton-Bishop J., Stratigos A.J. (2016). Diagnosis and Treatment of Melanoma. European Consensus-Based Interdisciplinary Guideline—Update 2016. Eur. J. Cancer.

[13] Wada-Ohno M., Ito T., Furue M. (2019). Adjuvant Therapy for Melanoma. Curr. Treat. Options Oncol..

[14] Seth R., Messersmith H., Kaur V., Kirkwood J.M., Kudchadkar R., McQuade J.L., Provenzano A., Swami U., Weber J., Alluri K.C. (2020). Systemic Therapy for Melanoma: ASCO Guideline. J. Clin. Oncol..

[15] Skudalski L., Waldman R., Kerr P.E., Grant-Kels J.M. (2022). Melanoma: An Update on Systemic Therapies. J. Am. Acad. Dermatol..

[16] Naidoo C., Kruger C.A., Abrahamse H. (2018). Photodynamic Therapy for Metastatic Melanoma Treatment: A Review. Technol. Cancer Res. Treat..

[17] Domingues B., Lopes J.M., Soares P., Pópulo H. (2018). Melanoma Treatment in Review. Immunotargets Ther..

[18] Castro K.A.D.F., Costa L.D., Guieu S., Biazzotto J.C., da Neves M.G.P.M.S., Faustino M.A.F., da Silva R.S., Tomé A.C. (2020). Photodynamic Treatment of Melanoma Cells Using Aza-Dipyrromethenes as Photosensitizers. Photochem. Photobiol. Sci..

[19] Jung E., Shim I., An J., Ji M.S., Jangili P., Chi S.-G., Kim J.S. (2021). Phenylthiourea-Conjugated BODIPY as an Efficient Photosensitizer for Tyrosinase-Positive Melanoma-Targeted Photodynamic Therapy. ACS Appl. Bio Mater..

[20] Baldea I., Olteanu D.E., Bolfa P., Ion R.M., Decea N., Cenariu M., Banciu M., Sesarman A.V., Filip A.G. (2015). Efficiency of Photodynamic Therapy on WM35 Melanoma with Synthetic Porphyrins: Role of Chemical Structure, Intracellular Targeting and Antioxidant Defense. J. Photochem. Photobiol. B Biol..

[21] Li X., Lee S., Yoon J. (2018). Supramolecular Photosensitizers Rejuvenate Photodynamic Therapy. Chem. Soc. Rev..

[22] Kessel D. (2019). Photodynamic Therapy: A Brief History. J. Clin. Med..

[23] Dougherty T.J., Kaufman J.E., Goldfarb A., Weishaupt K.R., Boyle D., Mittleman A. (1978). Photoradiation Therapy for the Treatment of Malignant Tumors1. Cancer Res..

[24] Gunaydin G., Gedik M.E., Ayan S. (2021). Photodynamic Therapy—Current Limitations and Novel Approaches. Front. Chem..

[25] Lopes J., Rodrigues C.M.P., Gaspar M.M., Reis C.P. (2022). How to Treat Melanoma? The Current Status of Innovative Nanotechnological Strategies and the Role of Minimally Invasive Approaches like PTT and PDT. Pharmaceutics.

[26] Zhang P., Han T., Xia H., Dong L., Chen L., Lei L. (2022). Advances in Photodynamic Therapy Based on Nanotechnology and Its Application in Skin Cancer. Front. Oncol..

[27] Adnane F., El-Zayat E., Fahmy H.M. (2022). The Combinational Application of Photodynamic Therapy and Nanotechnology in Skin Cancer Treatment: A Review. Tissue Cell.

[28] Zhang J., Jiang C., Figueiró Longo J.P., Azevedo R.B., Zhang H., Muehlmann L.A. (2018). An Updated Overview on the Development of New Photosensitizers for Anticancer Photodynamic Therapy. Acta Pharm. Sin. B.

[29] Liu W.-T., Wang H.-T., Yeh Y.-H., Wong T.-W. (2023). An Update on Recent Advances of Photodynamic Therapy for Primary Cutaneous Lymphomas. Pharmaceutics.

[30] Chauhan A., Gretz N. (2021). Role of Visible Light on Skin Melanocytes: A Systematic Review. Photochem. Photobiol..

[31] Al Khatib M., Harir M., Costa J., Baratto M., Schiavo I., Trabalzini L., Pollini S., Rossolini G., Basosi R., Pogni R. (2018). Spectroscopic Characterization of Natural Melanin from a Streptomyces Cyaneofuscatus Strain and Comparison with Melanin Enzymatically Synthesized by Tyrosinase and Laccase. Molecules.

[32] Madkhali N., Alqahtani H.R., Al-Terary S., Laref A., Hassib A. (2019). Control of Optical Absorption and Fluorescence Spectroscopies of Natural Melanin at Different Solution Concentrations. Opt. Quant. Electron..

[33] Micillo R., Panzella L., Iacomino M., Prampolini G., Cacelli I., Ferretti A., Crescenzi O., Koike K., Napolitano A., d’Ischia M. (2017). Eumelanin Broadband Absorption Develops from Aggregation-Modulated Chromophore Interactions under Structural and Redox Control. Sci. Rep..

[34] Sadiq I., Kollias N., Baqer A. (2019). Spectroscopic Observations on Human Pigmentation. Photodermatol. Photoimmunol. Photomed..

[35] Li X.-Y., Tan L.-C., Dong L.-W., Zhang W.-Q., Shen X.-X., Lu X., Zheng H., Lu Y.-G. (2020). Susceptibility and Resistance Mechanisms During Photodynamic Therapy of Melanoma. Front. Oncol..

[36] Chizenga E.P., Abrahamse H. (2020). Nanotechnology in Modern Photodynamic Therapy of Cancer: A Review of Cellular Resistance Patterns Affecting the Therapeutic Response. Pharmaceutics.

[37] Freitas L.F., Hamblin M.R., Anzengruber F., Perussi J.R., Ribeiro A.O., Martins V.C.A., Plepis A.M.G. (2017). Zinc Phthalocyanines Attached to Gold Nanorods for Simultaneous Hyperthermic and Photodynamic Therapies against Melanoma in Vitro. J. Photochem. Photobiol. B Biol..

[38] Vera R.E., Lamberti M.J., Rivarola V.A., Rumie Vittar N.B. (2015). Developing Strategies to Predict Photodynamic Therapy Outcome: The Role of Melanoma Microenvironment. Tumour Biol..

[39] Meira W.V., Heinrich T.A., Cadena S.M.S.C., Martinez G.R. (2017). Melanogenesis Inhibits Respiration in B16-F10 Melanoma Cells Whereas Enhances Mitochondrial Cell Content. Exp. Cell Res..

[40] Fu W., Wu Z., Zheng R., Yin N., Han F., Zhao Z., Dai M., Han D., Wang W., Niu L. (2022). Inhibition Mechanism of Melanin Formation Based on Antioxidant Scavenging of Reactive Oxygen Species. Analyst.

[41] Gomaa I., Sebak A., Afifi N., Abdel-Kader M. (2017). Liposomal Delivery of Ferrous Chlorophyllin: A Novel Third Generation Photosensitizer for in Vitro PDT of Melanoma. Photodiagn. Photodyn. Ther..

[42] Abrahamse H., Hamblin M.R. (2016). New Photosensitizers for Photodynamic Therapy. Biochem. J..

[43] Tavakkoli Yaraki M., Liu B., Tan Y.N. (2022). Emerging Strategies in Enhancing Singlet Oxygen Generation of Nano-Photosensitizers Toward Advanced Phototherapy. Nano-Micro Lett..

[44] Dougherty T.J., Grindey G.B., Fiel R., Weishaupt K.R., Boyle D.G. (1975). Photoradiation Therapy. II. Cure of Animal Tumors with Hematoporphyrin and Light. J. Natl. Cancer Inst..

[45] Escudero A., Carrillo-Carrión C., Carmen Castillejos M., Romero-Ben E., Rosales-Barrios C., Khiar N. (2021). Photodynamic Therapy: Photosensitizers and Nanostructures. Mater. Chem. Front..

[46] O’Connor A.E., Gallagher W.M., Byrne A.T. (2009). Porphyrin and Nonporphyrin Photosensitizers in Oncology: Preclinical and Clinical Advances in Photodynamic Therapy. Photochem. Photobiol..

[47] Zhu S., Tian R., Antaris A.L., Chen X., Dai H. (2019). Near-Infrared-II Molecular Dyes for Cancer Imaging and Surgery. Adv. Mater..

[48] Kwiatkowski S., Knap B., Przystupski D., Saczko J., Kędzierska E., Knap-Czop K., Kotlińska J., Michel O., Kotowski K., Kulbacka J. (2018). Photodynamic Therapy—Mechanisms, Photosensitizers and Combinations. Biomed. Pharmacother..

[49] Boscencu R., Radulea N., Manda G., Machado I.F., Socoteanu R.P., Lupuliasa D., Burloiu A.M., Mihai D.P., Ferreira L.F.V. (2023). Porphyrin Macrocycles: General Properties and Theranostic Potential. Molecules.

[50] McFarland S.A., Mandel A., Dumoulin-White R., Gasser G. (2020). Metal-Based Photosensitizers for Photodynamic Therapy: The Future of Multimodal Oncology?. Curr. Opin. Chem. Biol..

[51] Kou J., Dou D., Yang L. (2017). Porphyrin Photosensitizers in Photodynamic Therapy and its Applications. Oncotarget.

[52] Gunaydin G., Gedik M.E., Ayan S. (2021). Photodynamic Therapy for the Treatment and Diagnosis of Cancer–A Review of the Current Clinical Status. Front. Chem..

[53] Linares I.A.P., Martinelli L.P., Moritz M.N.O., Selistre-de-Araujo H.S., De Oliveira K.T., Rodrigues Perussi J. (2022). Cytotoxicity of Structurally-Modified Chlorins Aimed for Photodynamic Therapy Applications. J. Photochem. Photobiol. A Chem..

[54] Brilkina A.A., Dubasova L.V., Sergeeva E.A., Pospelov A.J., Shilyagina N.Y., Shakhova N.M., Balalaeva I.V. (2019). Photobiological Properties of Phthalocyanine Photosensitizers Photosens, Holosens and Phthalosens: A Comparative in Vitro Analysis. J. Photochem. Photobiol. B Biol..

[55] Setaro F., Wennink J.W.H., Mäkinen P.I., Holappa L., Trohopoulos P.N., Ylä-Herttuala S., Van Nostrum C.F., De La Escosura A., Torres T. (2020). Amphiphilic Phthalocyanines in Polymeric Micelles: A Supramolecular Approach toward Efficient Third-Generation Photosensitizers. J. Mater. Chem. B.

[56] Fekrazad R., Nejat A., Kalhori K.A.M. (2017). Antimicrobial Photodynamic Therapy with Nanoparticles Versus Conventional Photosensitizer in Oral Diseases. Nanostructures for Antimicrobial Therapy.

[57] Mathai S., Smith T.A., Ghiggino K.P. (2007). Singlet Oxygen Quantum Yields of Potential Porphyrin-Based Photosensitisers for Photodynamic Therapy. Photochem. Photobiol. Sci..

[58] Nishimura T., Hara K., Honda N., Okazaki S., Hazama H., Awazu K. (2020). Determination and Analysis of Singlet Oxygen Quantum Yields of Talaporfin Sodium, Protoporphyrin IX, and Lipidated Protoporphyrin IX Using near-Infrared Luminescence Spectroscopy. Lasers Med. Sci..

[59] Myrzakhmetov B., Arnoux P., Mordon S., Acherar S., Tsoy I., Frochot C. (2021). Photophysical Properties of Protoporphyrin IX, Pyropheophorbide-a, and Photofrin^®^ in Different Conditions. Pharmaceuticals.

[60] Monteiro C.J.P., Pereira M.M., Azenha M.E., Burrows H.D., Serpa C., Arnaut L.G., Tapia M.J., Sarakha M., Wong-Wah-Chung P., Navaratnam S. (2005). A Comparative Study of Water Soluble 5,10,15,20-Tetrakis(2,6-Dichloro-3-Sulfophenyl)Porphyrin and Its Metal Complexes as Efficient Sensitizers for Photodegradation of Phenols. Photochem. Photobiol. Sci..

[61] Deda D.K., Pavani C., Caritá E., Baptista M.S., Toma H.E., Araki K. (2012). Correlation of Photodynamic Activity and Singlet Oxygen Quantum Yields in Two Series of Hydrophobic Monocationic Porphyrins. J. Porphyr. Phthalocyanines.

[62] Vakrat-Haglili Y., Weiner L., Brumfeld V., Brandis A., Salomon Y., Mcllroy B., Wilson B.C., Pawlak A., Rozanowska M., Sarna T. (2005). The Microenvironment Effect on the Generation of Reactive Oxygen Species by Pd−Bacteriopheophorbide. J. Am. Chem. Soc..

[63] Macpherson A.N., Kessel D., Morgan A.R., Munro I., Truscott T.G. (1990). A Photophysical Study of Some Purpurins. Faraday Trans..

[64] Staicu A., Smarandache A., Pascu A., Pascu M.L. (2017). Photophysics of Covalently Functionalized Single Wall Carbon Nanotubes with Verteporfin. Appl. Surf. Sci..

[65] Clement S., Sobhan M., Deng W., Camilleri E., Goldys E.M. (2017). Nanoparticle-Mediated Singlet Oxygen Generation from Photosensitizers. J. Photochem. Photobiol. A Chem..

[66] Wilkinson F., Helman W.P., Ross A.B. (1993). Quantum Yields for the Photosensitized Formation of the Lowest Electronically Excited Singlet State of Molecular Oxygen in Solution. J. Phys. Chem. Ref. Data.

[67] Zenkevich E., Sagun E., Knyukshto V., Shulga A., Mironov A., Efremova O., Bonnett R., Songca S.P., Kassem M. (1996). Photophysical and Photochemical Properties of Potential Porphyrin and Chlorin Photosensitizers for PDT. J. Photochem. Photobiol. B Biol..

[68] Demirbaş Ü., Ömeroğlu İ., Akçay H.T., Durmuş M., Kantekin H. (2021). Synthesis, Characterization, Photophysical and Photochemical Properties of Peripherally Tetra Benzodioxane Substituted Metal-Free Phthalocyanine and its Zinc(II) and Magnesium(II) Derivatives. J. Mol. Struct..

[69] Braslavsky S.E., Müller M., Mártire D.O., Pörting S., Bertolotti S.G., Chakravorti S., Koç-Weier G., Knipp B., Schaffner K. (1997). Photophysical Properties of Porphycene Derivatives (18 π Porphyrinoids). J. Photochem. Photobiol. B Biol..

[70] Masiera N., Ostapko J., Gorski A., Bojarska A., Gawryszewska I., Sadowy E., Hryniewicz W., Waluk J. (2020). Photoeradication of Bacteria with Porphycenes: Substituent Effects on the Efficiency. Eur. J. Med. Chem..

[71] Salice P., Arnbjerg J., Pedersen B.W., Toftegaard R., Beverina L., Pagani G.A., Ogilby P.R. (2010). Photophysics of Squaraine Dyes: Role of Charge-Transfer in Singlet Oxygen Production and Removal. J. Phys. Chem. A.

[72] Avirah R.R., Jayaram D.T., Adarsh N., Ramaiah D. (2012). Squaraine Dyes in PDT: From Basic Design to in Vivo Demonstration. Org. Biomol. Chem..

[73] Zhao X., Yao Q., Long S., Chi W., Yang Y., Tan D., Liu X., Huang H., Sun W., Du J. (2021). An Approach to Developing Cyanines with Simultaneous Intersystem Crossing Enhancement and Excited-State Lifetime Elongation for Photodynamic Antitumor Metastasis. J. Am. Chem. Soc..

[74] Lange N., Szlasa W., Saczko J., Chwiłkowska A. (2021). Potential of Cyanine Derived Dyes in Photodynamic Therapy. Pharmaceutics.

[75] DeRosa M. (2002). Photosensitized Singlet Oxygen and Its Applications. Coord. Chem. Rev..

[76] Shahinyan G.A., Amirbekyan A.Y., Markarian S.A. (2019). Photophysical Properties of Methylene Blue in Water and in Aqueous Solutions of Dimethylsulfoxide. Spectrochim. Acta Part A Mol. Biomol. Spectrosc..

[77] Sofyan N., Situmorang F.W., Ridhova A., Yuwono A.H., Udhiarto A. (2018). Visible Light Absorption and Photosensitizing Characteristics of Natural Yellow 3 Extracted from *Curcuma Longa* L. for Dye-Sensitized Solar Cell. IOP Conf. Ser. Earth Environ. Sci..

[78] Chignell C.F., Bilskj P., Reszka K.J., Motten A.G., Sik R.H., Dahl T.A. (1994). Spectral and Photochemical Properties of Curcumin. Photochem. Photobiol..

[79] Zlatić K., Ayouchia H.B.E., Anane H., Mihaljević B., Basarić N., Rohand T. (2020). Spectroscopic and Photophysical Properties of Mono- and Dithiosubstituted BODIPY Dyes. J. Photochem. Photobiol. A Chem..

[80] Zhang X.-F., Zhang G.Q., Zhu J. (2019). Methylated Unsymmetric BODIPY Compounds: Synthesis, High Fluorescence Quantum Yield and Long Fluorescence Time. J. Fluoresc..

[81] Sevieri M., Silva F., Bonizzi A., Sitia L., Truffi M., Mazzucchelli S., Corsi F. (2020). Indocyanine Green Nanoparticles: Are They Compelling for Cancer Treatment?. Front. Chem..

[82] Dhaini B., Wagner L., Moinard M., Daouk J., Arnoux P., Schohn H., Schneller P., Acherar S., Hamieh T., Frochot C. (2022). Importance of Rose Bengal Loaded with Nanoparticles for Anti-Cancer Photodynamic Therapy. Pharmaceuticals.

[83] Demartis S., Obinu A., Gavini E., Giunchedi P., Rassu G. (2021). Nanotechnology-Based Rose Bengal: A Broad-Spectrum Biomedical Tool. Dye. Pigment..

[84] Zheng Y., Ye J., Li Z., Chen H., Gao Y. (2021). Recent Progress in Sono-Photodynamic Cancer Therapy: From Developed New Sensitizers to Nanotechnology-Based Efficacy-Enhancing Strategies. Acta Pharm. Sin. B.

[85] de Andrade G.P., de Souza T.F., Cerchiaro G., Pinhal M.A., Ribeiro A.O., Girão M.J. (2021). Hypericin in Photobiological Assays: An Overview. Photodiagn. Photodyn. Ther..

[86] Mfouo-Tynga I.S., Dias L.D., Inada N.M., Kurachi C. (2021). Features of Third Generation Photosensitizers Used in Anticancer Photodynamic Therapy: Review. Photodiagn. Photodyn. Ther..

[87] Chen J., Fan T., Xie Z., Zeng Q., Xue P., Zheng T., Chen Y., Luo X., Zhang H. (2020). Advances in Nanomaterials for Photodynamic Therapy Applications: Status and Challenges. Biomaterials.

[88] Abrahamse H., Kruger C.A., Kadanyo S., Mishra A. (2017). Nanoparticles for Advanced Photodynamic Therapy of Cancer. Photomed. Laser Surg..

[89] Chen S., Hao X., Liang X., Zhang Q., Zhang C., Zhou G., Shen S., Jia G., Zhang J. (2016). Inorganic Nanomaterials as Carriers for Drug Delivery. J. Biomed. Nanotechnol..

[90] Bertrand N., Wu J., Xu X., Kamaly N., Farokhzad O.C. (2014). Cancer Nanotechnology: The Impact of Passive and Active Targeting in the Era of Modern Cancer Biology. Adv. Drug Deliv. Rev..

[91] Wang H., Tran T.T., Duong K.T., Nguyen T., Le U.M. (2022). Options of Therapeutics and Novel Delivery Systems of Drugs for the Treatment of Melanoma. Mol. Pharm..

[92] Yao Y., Zhou Y., Liu L., Xu Y., Chen Q., Wang Y., Wu S., Deng Y., Zhang J., Shao A. (2020). Nanoparticle-Based Drug Delivery in Cancer Therapy and Its Role in Overcoming Drug Resistance. Front. Mol. Biosci..

[93] Pellosi D.S., De Jesus P.D.C.C., Tedesco A.C. (2017). Spotlight on the Delivery of Photosensitizers: Different Approaches for Photodynamic-Based Therapies. Expert Opin. Drug Deliv..

[94] Gamaleia N.F., Shton I.O. (2015). Gold Mining for PDT: Great Expectations from Tiny Nanoparticles. Photodiagn. Photodyn. Ther..

[95] Ramanunny A.K., Wadhwa S., Gulati M., Singh S.K., Kapoor B., Dureja H., Chellappan D.K., Anand K., Dua K., Khursheed R. (2021). Nanocarriers for Treatment of Dermatological Diseases: Principle, Perspective and Practices. Eur. J. Pharmacol..

[96] Yu B., Tai H.C., Xue W., Lee L.J., Lee R.J. (2010). Receptor-Targeted Nanocarriers for Therapeutic Delivery to Cancer. Mol. Membr. Biol..

[97] Hong E.J., Choi D.G., Shim M.S. (2016). Targeted and Effective Photodynamic Therapy for Cancer Using Functionalized Nanomaterials. Acta Pharm. Sin. B.

[98] Zhao J., Gao N., Xu J., Zhu X., Ling G., Zhang P. (2023). Novel Strategies in Melanoma Treatment Using Silver Nanoparticles. Cancer Lett..

[99] Malindi Z., Barth S., Abrahamse H. (2022). The Potential of Antibody Technology and Silver Nanoparticles for Enhancing Photodynamic Therapy for Melanoma. Biomedicines.

[100] Marzi M., Osanloo M., Vakil M.K., Mansoori Y., Ghasemian A., Dehghan A., Zarenezhad E. (2022). Applications of Metallic Nanoparticles in the Skin Cancer Treatment. BioMed Res. Int..

[101] Wang P., Tang H., Zhang P. (2016). Plasmonic Nanoparticle-Based Hybrid Photosensitizers with Broadened Excitation Profile for Photodynamic Therapy of Cancer Cells. Sci. Rep..

[102] Vankayala R., Lin C.-C., Kalluru P., Chiang C.-S., Hwang K.C. (2014). Gold Nanoshells-Mediated Bimodal Photodynamic and Photothermal Cancer Treatment Using Ultra-Low Doses of near Infra-Red Light. Biomaterials.

[103] Singh S.K., Mazumder S., Vincy A., Hiremath N., Kumar R., Banerjee I., Vankayala R. (2023). Review of Photoresponsive Plasmonic Nanoparticles That Produce Reactive Chemical Species for Photodynamic Therapy of Cancer and Bacterial Infections. ACS Appl. Nano Mater..

[104] Nkune N.W., Abrahamse H. (2021). Nanoparticle-Based Drug Delivery Systems for Photodynamic Therapy of Metastatic Melanoma: A Review. Int. J. Mol. Sci..

[105] Pivetta T.P., Botteon C.E.A., Ribeiro P.A., Marcato P.D., Raposo M. (2021). Nanoparticle Systems for Cancer Phototherapy: An Overview. Nanomaterials.

[106] Yang Y.-L., Lin K., Yang L. (2021). Progress in Nanocarriers Codelivery System to Enhance the Anticancer Effect of Photodynamic Therapy. Pharmaceutics.

[107] Baldea I., Giurgiu L., Teacoe I.D., Olteanu D.E., Olteanu F.C., Clichici S., Filip G.A. (2019). Photodynamic Therapy in Melanoma—Where Do We Stand?. Curr. Med. Chem..

[108] Nowak-Sliwinska P., Karocki A., Elas M., Pawlak A., Stochel G., Urbanska K. (2006). Verteporfin, Photofrin II, and Merocyanine 540 as PDT Photosensitizers against Melanoma Cells. Biochem. Biophys. Res. Commun..

[109] Hamblin M.R., Chiang L.Y., Lakshmanan S., Huang Y.-Y., Garcia-Diaz M., Karimi M., de Souza Rastelli A.N., Chandran R. (2015). Nanotechnology for Photodynamic Therapy: A Perspective from the Laboratory of Dr. Michael R. Hamblin in the Wellman Center for Photomedicine at Massachusetts General Hospital and Harvard Medical School. Nanotechnol. Rev..

[110] Harada Y., Murayama Y., Takamatsu T., Otsuji E., Tanaka H. (2022). 5-Aminolevulinic Acid-Induced Protoporphyrin IX Fluorescence Imaging for Tumor Detection: Recent Advances and Challenges. Int. J. Mol. Sci..

[111] Traylor J.I., Pernik M.N., Sternisha A.C., McBrayer S.K., Abdullah K.G. (2021). Molecular and Metabolic Mechanisms Underlying Selective 5-Aminolevulinic Acid-Induced Fluorescence in Gliomas. Cancers.

[112] Lou L., Zhou S., Tan S., Xiang M., Wang W., Yuan C., Gao L., Xiao Q. (2023). Amplifying the Efficacy of ALA-Based Prodrugs for Photodynamic Therapy Using Nanotechnology. Front. Pharmacol..

[113] Mohammadi Z., Sazgarnia A., Rajabi O., Soudmand S., Esmaily H., Sadeghi H.R. (2013). An in Vitro Study on the Photosensitivity of 5-Aminolevulinic Acid Conjugated Gold Nanoparticles. Photodiagn. Photodyn. Ther..

[114] Ma X., Qu Q., Zhao Y. (2015). Targeted Delivery of 5-Aminolevulinic Acid by Multifunctional Hollow Mesoporous Silica Nanoparticles for Photodynamic Skin Cancer Therapy. ACS Appl. Mater. Interfaces.

[115] Harmatys K.M., Musso A.J., Clear K.J., Smith B.D. (2016). Small Molecule Additive Enhances Cell Uptake of 5-Aminolevulinic Acid and Conversion to Protoporphyrin IX. Photochem. Photobiol. Sci..

[116] Li A., Liang C., Xu L., Wang Y., Liu W., Zhang K., Liu J., Shi J. (2021). Boosting 5-ALA-Based Photodynamic Therapy by a Liposomal Nanomedicine through Intracellular Iron Ion Regulation. Acta Pharm. Sin. B.

[117] Velasquez J., Wray A.A. (2023). Deferoxamine. StatPearls.

[118] Li Y., Ruan S., Guo J., He Z., Xia Q., Wu T., Wang Z., Li Z., Hu H., Jing Q. (2022). B16F10 Cell Membrane-Based Nanovesicles for Melanoma Therapy Are Superior to Hyaluronic Acid-Modified Nanocarriers. Mol. Pharm..

[119] da Silva D.B., da Silva C.L., Davanzo N.N., da Silva Souza R., Correa R.J., Tedesco A.C., Riemma Pierre M.B. (2021). Protoporphyrin IX (PpIX) Loaded PLGA Nanoparticles for Topical Photodynamic Therapy of Melanoma Cells. Photodiagn. Photodyn. Ther..

[120] Vadarevu H., Juneja R., Lyles Z., Vivero-Escoto J.L. (2021). Light-Activated Protoporphyrin IX-Based Polysilsesquioxane Nanoparticles Induce Ferroptosis in Melanoma Cells. Nanomaterials.

[121] Rizzi M., Tonello S., Estevão B.M., Gianotti E., Marchese L., Renò F. (2017). Verteporfin Based Silica Nanoparticle for in Vitro Selective Inhibition of Human Highly Invasive Melanoma Cell Proliferation. J. Photochem. Photobiol. B Biol..

[122] Zhang H., Ramakrishnan S.K., Triner D., Centofanti B., Maitra D., Győrffy B., Sebolt-Leopold J.S., Dame M.K., Varani J., Brenner D.E. (2015). Tumor-Selective Proteotoxicity of Verteporfin Inhibits Colon Cancer Progression Independently of YAP1. Sci. Signal.

[123] Argyo C., Weiss V., Bräuchle C., Bein T. (2014). Multifunctional Mesoporous Silica Nanoparticles as a Universal Platform for Drug Delivery. Chem. Mater..

[124] Nistorescu S., Udrea A.-M., Badea M.A., Lungu I., Boni M., Tozar T., Dumitrache F., Maraloiu V.-A., Popescu R.G., Fleaca C. (2021). Low Blue Dose Photodynamic Therapy with Porphyrin-Iron Oxide Nanoparticles Complexes: In Vitro Study on Human Melanoma Cells. Pharmaceutics.

[125] Balas M., Nistorescu S., Badea M.A., Dinischiotu A., Boni M., Dinache A., Smarandache A., Udrea A.-M., Prepelita P., Staicu A. (2023). Photodynamic Activity of TMPyP4/TiO_2_ Complex under Blue Light in Human Melanoma Cells: Potential for Cancer-Selective Therapy. Pharmaceutics.

[126] Chen Z.-A., Kuthati Y., Kankala R.K., Chang Y.-C., Liu C.-L., Weng C.-F., Mou C.-Y., Lee C.-H. (2015). Encapsulation of Palladium Porphyrin Photosensitizer in Layered Metal Oxide Nanoparticles for Photodynamic Therapy against Skin Melanoma. Sci. Technol. Adv. Mater..

[127] Ogawara K., Shiraishi T., Araki T., Watanabe T., Ono T., Higaki K. (2016). Efficient Anti-Tumor Effect of Photodynamic Treatment with Polymeric Nanoparticles Composed of Polyethylene Glycol and Polylactic Acid Block Copolymer Encapsulating Hydrophobic Porphyrin Derivative. Eur. J. Pharm. Sci..

[128] Lee K.L., Carpenter B.L., Wen A.M., Ghiladi R.A., Steinmetz N.F. (2016). High Aspect Ratio Nanotubes Formed by Tobacco Mosaic Virus for Delivery of Photodynamic Agents Targeting Melanoma. ACS Biomater. Sci. Eng..

[129] Zhou Y., Liang X., Dai Z. (2016). Porphyrin-Loaded Nanoparticles for Cancer Theranostics. Nanoscale.

[130] Chlorin (CHEBI:36303). https://www.ebi.ac.uk/chebi/searchId.do?chebiId=CHEBI:36303.

[131] Mbakidi J.P., Drogat N., Granet R., Ouk T.-S., Ratinaud M.-H., Rivière E., Verdier M., Sol V. (2013). Hydrophilic Chlorin-Conjugated Magnetic Nanoparticles—Potential Anticancer Agent for the Treatment of Melanoma by PDT. Bioorg. Med. Chem. Lett..

[132] Chen Z., Feng T., Shen J., Karges J., Jin C., Zhao Y., Ji L., Chao H. (2022). A Mitochondria-Localized Iridium(III)–Chlorin E6 Conjugate for Synergistic Sonodynamic and Two-Photon Photodynamic Therapy against Melanoma. Inorg. Chem. Front..

[133] Baskaran R., Lee J., Yang S.-G. (2018). Clinical Development of Photodynamic Agents and Therapeutic Applications. Biomater. Res..

[134] Liu R., Gao Y., Liu N., Suo Y. (2021). Nanoparticles Loading Porphyrin Sensitizers in Improvement of Photodynamic Therapy for Ovarian Cancer. Photodiagn. Photodyn. Ther..

[135] Gaio E., Conte C., Esposito D., Reddi E., Quaglia F., Moret F. (2020). CD44 Targeting Mediated by Polymeric Nanoparticles and Combination of Chlorine TPCS2a-PDT and Docetaxel-Chemotherapy for Efficient Killing of Breast Differentiated and Stem Cancer Cells In Vitro. Cancers.

[136] Montaseri H., Kruger C.A., Abrahamse H. (2020). Review: Organic Nanoparticle Based Active Targeting for Photodynamic Therapy Treatment of Breast Cancer Cells. Oncotarget.

[137] Vignion-Dewalle A.-S., Baert G., Thecua E., Vicentini C., Mortier L., Mordon S. (2017). Photodynamic Therapy for Actinic Keratosis: Is the European Consensus Protocol for Daylight PDT Superior to Conventional Protocol for Aktilite CL 128 PDT?. J. Photochem. Photobiol. B Biol..

[138] Hak A., Ali M.S., Sankaranarayanan S.A., Shinde V.R., Rengan A.K. (2023). Chlorin E6: A Promising Photosensitizer in Photo-Based Cancer Nanomedicine. ACS Appl. Bio Mater..

[139] Kulichenko A., Farrakhova D.S., Yakovlev D.V., Maklygina Y.S., Shiryaev A.A., Loschenov V.B. (2021). Fluorescence Diagnostics and Photodynamic Therapy of Squamous Cell Carcinoma of the Lateral Surface of the Tongue Using the Photosensitizer Chlorin E6 by Spectroscopic Video Fluorescence Methods. J. Phys. Conf. Ser..

[140] Li T.-F., Xu H.-Z., Xu Y.-H., Yu T.-T., Tang J.-M., Li K., Wang C., Peng X.-C., Li Q.-R., Sang X.-Y. (2021). Efficient Delivery of Chlorin E6 by Polyglycerol-Coated Iron Oxide Nanoparticles with Conjugated Doxorubicin for Enhanced Photodynamic Therapy of Melanoma. Mol. Pharm..

[141] Wang S., Liu H., Xin J., Rahmanzadeh R., Wang J., Yao C., Zhang Z. (2019). Chlorin-Based Photoactivable Galectin-3-Inhibitor Nanoliposome for Enhanced Photodynamic Therapy and NK Cell-Related Immunity in Melanoma. ACS Appl. Mater. Interfaces.

[142] Zhu Y., Xue J., Chen W., Bai S., Zheng T., He C., Guo Z., Jiang M., Du G., Sun X. (2020). Albumin-Biomineralized Nanoparticles to Synergize Phototherapy and Immunotherapy against Melanoma. J. Control. Release.

[143] Chu M., Li H., Wu Q., Wo F., Shi D. (2014). Pluronic-Encapsulated Natural Chlorophyll Nanocomposites for in Vivo Cancer Imaging and Photothermal/Photodynamic Therapies. Biomaterials.

[144] Ash C., Dubec M., Donne K., Bashford T. (2017). Effect of Wavelength and Beam Width on Penetration in Light-Tissue Interaction Using Computational Methods. Lasers Med. Sci..

[145] Wright J.D. (2001). Phthalocyanines. Encyclopedia of Materials: Science and Technology.

[146] Staicu A., Pascu A., Nuta A., Sorescu A., Ratitoiu V., Pascu M.L. (2013). Studies About Phthalocyanine Photosensitizers to be Used in Photodynamic Therapy. Rom. Rep. Phys..

[147] Bolfarini G.C., Siqueira-Moura M.P., Demets G.J.F., Morais P.C., Tedesco A.C. (2012). In Vitro Evaluation of Combined Hyperthermia and Photodynamic Effects Using Magnetoliposomes Loaded with Cucurbituril Zinc Phthalocyanine Complex on Melanoma. J. Photochem. Photobiol. B Biol..

[148] Do Reis S.R.R., Helal-Neto E., Da Silva De Barros A.O., Pinto S.R., Portilho F.L., De Oliveira Siqueira L.B., Alencar L.M.R., Dahoumane S.A., Alexis F., Ricci-Junior E. (2021). Dual Encapsulated Dacarbazine and Zinc Phthalocyanine Polymeric Nanoparticle for Photodynamic Therapy of Melanoma. Pharm. Res..

[149] Camerin M., Moreno M., Marín M.J., Schofield C.L., Chambrier I., Cook M.J., Coppellotti O., Jori G., Russell D.A. (2016). Delivery of a Hydrophobic Phthalocyanine Photosensitizer Using PEGylated Gold Nanoparticle Conjugates for the in Vivo Photodynamic Therapy of Amelanotic Melanoma. Photochem. Photobiol. Sci..

[150] Camerin M., Magaraggia M., Soncin M., Jori G., Moreno M., Chambrier I., Cook M.J., Russell D.A. (2010). The in Vivo Efficacy of Phthalocyanine–Nanoparticle Conjugates for the Photodynamic Therapy of Amelanotic Melanoma. Eur. J. Cancer.

[151] Goto P.L., Siqueira-Moura M.P., Tedesco A.C. (2017). Application of Aluminum Chloride Phthalocyanine-Loaded Solid Lipid Nanoparticles for Photodynamic Inactivation of Melanoma Cells. Int. J. Pharm..

[152] Almeida E.D.P., Dipieri L.V., Rossetti F.C., Marchetti J.M., Bentley M.V.L.B., Nunes R.D.S., Sarmento V.H.V., Valerio M.E.G., Rodrigues Júnior J.J., Montalvão M.M. (2018). Skin Permeation, Biocompatibility and Antitumor Effect of Chloroaluminum Phthalocyanine Associated to Oleic Acid in Lipid Nanoparticles. Photodiagn. Photodyn. Ther..

[153] Mello V.C., Araújo V.H.S., De Paiva K.L.R., Simões M.M., Marques D.C., Da Silva Costa N.R., De Souza I.F., Da Silva P.B., Santos I., Almeida R. (2022). Development of New Natural Lipid-Based Nanoparticles Loaded with Aluminum-Phthalocyanine for Photodynamic Therapy against Melanoma. Nanomaterials.

[154] D’Alessandro S., Priefer R. (2020). Non-Porphyrin Dyes Used as Photosensitizers in Photodynamic Therapy. J. Drug Deliv. Sci. Technol..

[155] Liu H., Yin J., Xing E., Du Y., Su Y., Feng Y., Meng S. (2021). Halogenated Cyanine Dyes for Synergistic Photodynamic and Photothermal Therapy. Dye. Pigment..

[156] Staurenghi G., Bottoni F., Giani A., Ryan S.J., Sadda S.R., Hinton D.R., Schachat A.P., Sadda S.R., Wilkinson C.P., Wiedemann P., Schachat A.P. (2013). Chapter 2—Clinical Applications of Diagnostic Indocyanine Green Angiography. Retina.

[157] Olubiyi O.I., Lu F.-K., Calligaris D., Jolesz F.A., Agar N.Y., Golby A.J. (2015). Chapter 17—Advances in Molecular Imaging for Surgery. Image-Guided Neurosurgery.

[158] Hu H., Chen J., Yang H., Huang X., Wu H., Wu Y., Li F., Yi Y., Xiao C., Li Y. (2019). Potentiating Photodynamic Therapy of ICG-Loaded Nanoparticles by Depleting GSH with PEITC. Nanoscale.

[159] Tang J., Zhou H., Hou X., Wang L., Li Y., Pang Y., Chen C., Jiang G., Liu Y. (2018). Enhanced Anti-Tumor Efficacy of Temozolomide-Loaded Carboxylated Poly(Amido-Amine) Combined with Photothermal/Photodynamic Therapy for Melanoma Treatment. Cancer Lett..

[160] Jin Y.-J., Termsarasab U., Ko S.-H., Shim J.-S., Chong S., Chung S.-J., Shim C.-K., Cho H.-J., Kim D.-D. (2012). Hyaluronic Acid Derivative-Based Self-Assembled Nanoparticles for the Treatment of Melanoma. Pharm. Res..

[161] Campu A., Focsan M., Lerouge F., Borlan R., Tie L., Rugina D., Astilean S. (2020). ICG-Loaded Gold Nano-Bipyramids with NIR Activatable Dual PTT-PDT Therapeutic Potential in Melanoma Cells. Colloids Surf. B Biointerfaces.

[162] Mondal S., Nguyen T.P., Pham V.H., Hoang G., Manivasagan P., Kim M.H., Nam S.Y., Oh J. (2020). Hydroxyapatite Nano Bioceramics Optimized 3D Printed Poly Lactic Acid Scaffold for Bone Tissue Engineering Application. Ceram. Int..

[163] Wen L., Hyoju R., Wang P., Shi L., Li C., Li M., Wang X. (2021). Hydrogen-Peroxide-Responsive Protein Biomimetic Nanoparticles for Photothermal-Photodynamic Combination Therapy of Melanoma. Lasers Surg. Med..

[164] Lee E.-H., Lim S.-J., Lee M.-K. (2019). Chitosan-Coated Liposomes to Stabilize and Enhance Transdermal Delivery of Indocyanine Green for Photodynamic Therapy of Melanoma. Carbohydr. Polym..

[165] Han Y.-H., Kankala R., Wang S.-B., Chen A.-Z. (2018). Leveraging Engineering of Indocyanine Green-Encapsulated Polymeric Nanocomposites for Biomedical Applications. Nanomaterials.

[166] Sudhakar K., Fuloria S., Subramaniyan V., Sathasivam K.V., Azad A.K., Swain S.S., Sekar M., Karupiah S., Porwal O., Sahoo A. (2021). Ultraflexible Liposome Nanocargo as a Dermal and Transdermal Drug Delivery System. Nanomaterials.

[167] Alavi S., Haeri A., Dadashzadeh S. (2017). Utilization of Chitosan-Caged Liposomes to Push the Boundaries of Therapeutic Delivery. Carbohydr. Polym..

[168] Ali A., Ahmed S. (2018). A Review on Chitosan and Its Nanocomposites in Drug Delivery. Int. J. Biol. Macromol..

[169] Liao J., Wei X., Ran B., Peng J., Qu Y., Qian Z. (2017). Polymer Hybrid Magnetic Nanocapsules Encapsulating IR820 and PTX for External Magnetic Field-Guided Tumor Targeting and Multifunctional Theranostics. Nanoscale.

[170] Hou X., Tao Y., Li X., Pang Y., Yang C., Jiang G., Liu Y. (2020). CD44-Targeting Oxygen Self-Sufficient Nanoparticles for Enhanced Photodynamic Therapy Against Malignant Melanoma. Int. J. Nanomed..

[171] Gianotti E., Martins Estevão B., Cucinotta F., Hioka N., Rizzi M., Renò F., Marchese L. (2014). An Efficient Rose Bengal Based Nanoplatform for Photodynamic Therapy. Chem. Eur. J..

[172] Pednekar P.P., Godiyal S.C., Jadhav K.R., Kadam V.J. (2017). Mesoporous Silica Nanoparticles: A Promising Multifunctional Drug Delivery System. Nanostructures for Cancer Therapy.

[173] Bazylińska U., Wawrzyńczyk D., Szewczyk A., Kulbacka J. (2020). Engineering and Biological Assessment of Double Core Nanoplatform for Co-Delivery of Hybrid Fluorophores to Human Melanoma. J. Inorg. Biochem..

[174] Chen H.-J., Zhou X.-B., Wang A.-L., Zheng B.-Y., Yeh C.-K., Huang J.-D. (2018). Synthesis and Biological Characterization of Novel Rose Bengal Derivatives with Improved Amphiphilicity for Sono-Photodynamic Therapy. Eur. J. Med. Chem..

[175] Mohseni H., Imanparast A., Salarabadi S.S., Sazgarnia A. (2022). In Vitro Evaluation of the Intensifying Photodynamic Effect Due to the Presence of Plasmonic Hollow Gold Nanoshells Loaded with Methylene Blue on Breast and Melanoma Cancer Cells. Photodiagn. Photodyn. Ther..

[176] Magalhães J.A., Arruda D.C., Baptista M.S., Tada D.B. (2021). Co-Encapsulation of Methylene Blue and PARP-Inhibitor into Poly(Lactic-Co-Glycolic Acid) Nanoparticles for Enhanced PDT of Cancer. Nanomaterials.

[177] Gobo G.G., Piva H.L., Tedesco A.C., Primo F.L. (2022). Novel Quinizarin Spray-Dried Nanoparticles for Treating Melanoma with Photodynamic Therapy. Mater. Today Commun..

[178] Wang W., Wang L., Li Z., Xie Z. (2016). BODIPY-Containing Nanoscale Metal–Organic Frameworks for Photodynamic Therapy. Chem. Commun..

[179] Oh J.S., You Y., Park K.C., Gupta G., Kang D.-K., Lee C.Y. (2019). Toward an Efficient Photosensitizer for Photodynamic Therapy: Incorporating BODIPY into Porphyrinic Nanoscale MOFs through the Solvent-Assisted Ligand Incorporation. Dye. Pigment..

[180] De Morais F.A.P., De Oliveira A.C.V., Balbinot R.B., Lazarin-Bidóia D., Ueda-Nakamura T., De Oliveira Silva S., Da Silva Souza Campanholi K., Da Silva Junior R.C., Gonçalves R.S., Caetano W. (2022). Multifunctional Nanoparticles as High-Efficient Targeted Hypericin System for Theranostic Melanoma. Polymers.

[181] Ghazaeian M., Khorsandi K., Hosseinzadeh R., Naderi A., Abrahamse H. (2021). Curcumin–Silica Nanocomplex Preparation, Hemoglobin and DNA Interaction and Photocytotoxicity against Melanoma Cancer Cells. J. Biomol. Struct. Dyn..

[182] Sujai P.T., Joseph M.M., Karunakaran V., Saranya G., Adukkadan R.N., Shamjith S., Thomas R., Nair J.B., Swathi R.S., Maiti K.K. (2019). Biogenic Cluster-Encased Gold Nanorods as a Targeted Three-in-One Theranostic Nanoenvelope for SERS-Guided Photochemotherapy against Metastatic Melanoma. ACS Appl. Bio Mater..

[183] Benstead M., Mehl G.H., Boyle R.W. (2011). 4,4′-Difluoro-4-Bora-3a,4a-Diaza-s-Indacenes (BODIPYs) as Components of Novel Light Active Materials. Tetrahedron.

[184] Yu Z., Wang H., Chen Z., Dong X., Zhao W., Shi Y., Zhu Q. (2022). Discovery of an Amino Acid-Modified Near-Infrared Aza-BODIPY Photosensitizer as an Immune Initiator for Potent Photodynamic Therapy in Melanoma. J. Med. Chem..

[185] Kubin A., Wierrani F., Burner U., Alth G., Grunberger W. (2005). Hypericin—The Facts About a Controversial Agent. Curr. Pharm. Des..

